# The PGRS Domain of Mycobacterium tuberculosis PE_PGRS Protein Rv0297 Is Involved in Endoplasmic Reticulum Stress-Mediated Apoptosis through Toll-Like Receptor 4

**DOI:** 10.1128/mBio.01017-18

**Published:** 2018-06-19

**Authors:** Sonam Grover, Tarina Sharma, Yadvir Singh, Sakshi Kohli, Manjunath P., Aditi Singh, Torsten Semmler, Lothar H. Wieler, Karsten Tedin, Nasreen Z. Ehtesham, Seyed E. Hasnain

**Affiliations:** a JH Institute of Molecular Medicine, Jamia Hamdard, New Delhi, India; b Molecular Infection and Functional Biology Lab, Kusuma School of Biological Sciences, Indian Institute of Technology—Delhi, New Delhi, India; c Department of Molecular Biology, Max Planck Institute for Infection Biology, Berlin, Germany; d Centre for Infectious Disease and Research, Indian Institute of Science, Bangalore, India; e School of Biotechnology, Jawaharlal Nehru University, New Delhi, India; f Microbial Genomics Research Group, Robert Koch Institute, Berlin, Germany; g Centre for Infection Medicine, Institute of Microbiology and Epizootics, Berlin, Germany; h National Institute of Pathology, Safdarjung Hospital Campus, New Delhi, India; i Dr Reddy’s Institute of Life Sciences, University of Hyderabad Campus, Hyderabad, India; Vallabhbhai Patel Chest Institute

**Keywords:** calcium homeostasis, ER localization signal, granulomas, unfolded protein response

## Abstract

The genome of Mycobacterium tuberculosis, the causal organism of tuberculosis (TB), encodes a unique protein family known as the PE/PPE/PGRS family, present exclusively in the genus *Mycobacterium* and nowhere else in the living kingdom, with largely unexplored functions. We describe the functional significance of the PGRS domain of Rv0297, a member of this family. *In silico* analyses revealed the presence of intrinsically disordered stretches and putative endoplasmic reticulum (ER) localization signals in the PGRS domain of Rv0297 (Rv0297PGRS). The PGRS domain aids in ER localization, which was shown by infecting macrophage cells with *M. tuberculosis* and by overexpressing the protein by transfection in macrophage cells followed by activation of the unfolded protein response, as evident from increased expression of GRP78/GRP94 and CHOP/ATF4, leading to disruption of intracellular Ca^2+^ homeostasis and increased nitric oxide (NO) and reactive oxygen species (ROS) production. The consequent activation of the effector caspase-8 resulted in apoptosis of macrophages, which was Toll-like receptor 4 (TLR4) dependent. Administration of recombinant Rv0297PGRS (rRv0297PGRS) also exhibited similar effects. These results implicate a hitherto-unknown role of the PGRS domain of the PE_PGRS protein family in ER stress-mediated cell death through TLR4. Since this protein is already known to be present at later stages of infection in human granulomas it points to the possibility of it being employed by *M. tuberculosis* for its dissemination via an apoptotic mechanism.

## IMPORTANCE

Apoptosis is generally thought to be a defense mechanism in protecting the host against Mycobacterium tuberculosis in early stages of infection. However, apoptosis during later stages in lung granulomas may favor the bacterium in disseminating the disease. ER stress has been found to induce apoptosis in TB granulomas, in zones where apoptotic macrophages accumulate in mice and humans. In this study, we report ER stress-mediated apoptosis of host cells by the Rv0297-encoded PE_PGRS5 protein of M. tuberculosis exceptionally present in the pathogenic *Mycobacterium* genus. The PGRS domain of Rv0297 aids the protein in localizing to the ER and induces the unfolded protein response followed by apoptosis of macrophages. The effect of the Rv0297PGRS domain was found to be TLR4 dependent. This study presents novel insights on the strategies employed by M. tuberculosis to disseminate the disease.

## INTRODUCTION

Tuberculosis (TB) remains a major public health problem and is caused by infections with the pathogen Mycobacterium tuberculosis. According to the WHO 2015 TB report, 9.6 million new cases of TB infection were reported globally, with approximately 1.5 million deaths (1). One-third of the world’s population is estimated to be latently infected with *M. tuberculosis*. The problem is further compounded by the emergence of drug-resistant forms, HIV coinfections, and diabetes (2) and the lack of an effective vaccine.

Approximately 10% of the coding capacity of the *M. tuberculosis* genome is dedicated to the PE and PPE gene family members, so termed due to the occurrence of PE and PPE domains close to the N-terminal region (3–5). This family is present exclusively in the genus *Mycobacterium* and nowhere else in the living kingdom (4). Various PE/PPE proteins of *M. tuberculosis* have been reported to be expressed upon infection of macrophages and play crucial roles in virulence, antigenic diversity, and modulation of the host immune response (6–8). Numerous members of the PE gene family display several copies of polymorphic guanine-cytosine-rich sequences (PGRSs) at their C-terminal ends in the so-called PE_PGRS subfamily (9). In the past decade, there has been a growing interest in determining the role of PE_PGRS proteins in the pathophysiology of TB due to their limited presence in nonpathogenic mycobacteria (10). Various members of the PE_PGRS family stimulate strong T-cell responses and immune quorum sensing (3, 11). The PGRS domain of *M. tuberculosis* PE_PGRS33 (Rv1818c) is responsible for inducing humoral as well as cellular immune responses in humans, and there is also evidence for the presence of major histocompatibility complex class I (MHC-I)-restricted CD8^+^ T cells in mice, suggesting their highly immunogenic nature (12, 13). Mutations in the homologs of PE_PGRS62 and PE_PGRS30 in Mycobacterium marinum resulted in reduced persistence of these bacteria in granulomas (14). In addition, the Wag22 PE_PGRS antigen-mediated immune response was found to be involved in sustaining latent infection in a mouse model of chronic *M. tuberculosis* infection (15). PE_PGRS proteins have also been found at the cell surface of *M. tuberculosis* and found to affect its cell surface interactions (16). Their association with the cell wall and surface exposure also leads to trafficking of these proteins out of the mycobacterial phagosome into endocytic compartments. Few of the PE_PGRS proteins are exocytosed into the extracellular environment (17). Localization of Rv1818c to mitochondria resulted in induction of cell death (18) and was also shown to be involved in enhanced survival of Mycobacterium smegmatis in macrophages (11).

The stress pathway mediated through the endoplasmic reticulum (ER), also known as the unfolded protein response (UPR), is an alternative survival pathway which guards cells from the effects of the accumulation of misfolded/unfolded proteins (19). However, under certain situations the UPR can cause cell death via apoptosis, which has a significant role in the pathogenicity and survival of intracellular *M. tuberculosis* (20). Interestingly, ER stress is induced in specific areas of TB granulomas where apoptotic macrophages accumulate (21). ER stress-mediated apoptosis of host cells caused by mycobacterial proteins could be a potential mechanism, which has also been reported for two important TB vaccine candidate proteins, ESAT-6 and HBHA (22, 23). However, little is known about the effect of PE_PGRS proteins on granuloma formation and the cell death mechanism.

Proteomic analysis revealed the presence of a hypothetical protein, Rv0297, in lung granulomas 90 days postinfection (24). It is one of the T-cell antigens for which memory T cells are present in latently *M. tuberculosis*-infected individuals (25). Rv0297 is present in the region of the *M. tuberculosis*-complex-specific genomic island as revealed by horizontal gene transfer studies (26). It is also a part of the clusters of *M. tuberculosis* genes which are downregulated 4 h postinfection in human macrophages (THP-1) (27). However, the biological role of Rv0297 remains unknown. This study was designed to delineate the mechanism of action of Rv0297 on host cell death, particularly the role of its PGRS domain alone or in fusion with the PE domain. In contrast to previous reports on the involvement of the PGRS domain in the mitochondrion-mediated cell death pathway, here we show that the Rv0297PGRS domain is associated with ER stress-mediated apoptosis.

## RESULTS

### Multiple sequence alignments (MSAs) reveal amino acid sequence conservation within PE domains but variations in the PGRS domain.

Members of the PE/PPE family of *M. tuberculosis*, Rv1788 and two PE_PGRS proteins (Rv1818c and Rv0297), were selected for this study. MSAs of PE domains of Rv0297, Rv1788, and Rv1818c revealed a strong conservation in the first 57 residues among the three proteins. Alignment of Rv0297 and Rv1818c showed conservation of around 80 residues, some of which were present in the PE domain and the rest in the PGRS domain (see Fig. S2A in the supplemental material). Alignment of the PGRS domains of Rv0297 and Rv1818c revealed significantly higher variation of amino acid sequences between these two proteins (Fig. S2B).

10.1128/mBio.01017-18.1 FIG S1  Schematic of the cloning strategy and fusion constructs used. (A) Cloning was performed in pDsRed1C1 expression vector using the XhoI-BamHI site. (B) Graphic representation of the various fusion proteins constructed using this method. Download FIG S1, TIF file, 1.1 MB. Copyright © 2018 Grover et al. 2018 Grover et al. This content is distributed under the terms of the Creative Commons Attribution 4.0 International license.

10.1128/mBio.01017-18.2 FIG S2  Multiple sequence alignment (MSA) reveals amino acid sequence conservation with PE domains but variations in the PGRS domain. (A) MSA of PE domains of Rv0297, Rv1818c, and Rv1788. (B) MSA of PGRS domain Rv0297 and Rv1818c. MSA was performed using the T-Coffee alignment server (http://tcoffee.org). The MSA shows a high degree of amino acid sequence similarity in the PE domain compared to the PGRS domain. Download FIG S2, TIF file, 2.1 MB. Copyright © 2018 Grover et al. 2018 Grover et al. This content is distributed under the terms of the Creative Commons Attribution 4.0 International license.

Our initial experiments revealed that Rv0297PGRS alone or in fusion with the PE domain resulted in cell death (Fig. S3). When HEK293T cells were transfected with various constructs and stained with nuclear stain Hoechst 33342 (30 h and 48 h postinfection), those cells transfected with constructs harboring Rv0297PGRS showed rounding-off phenomena after 30 h (Fig. S3A). After 48 h, cells showed signs of cell death and disruption (Fig. S3B). To delineate the mechanism of action of Rv0297 and its possible role in the virulence of mycobacteria during infection in humans, we chose to examine the possible mechanism of cell death in murine macrophage cells.

10.1128/mBio.01017-18.3 FIG S3  Expression of the PGRS domain of Rv0297 leads to apoptosis as evident from cellular morphology. Cells were transfected with various constructs containing the Rv0297 PE domain (a), the Rv0297PGRS domain (b), the Rv0297 full-length protein with both PE and PGRS domains (c), the PE domain of Rv1788 fused with the PGRS domain of Rv0297 (d), and the PE domain of Rv1818c fused with the PGRS domain of Rv0297 (e), transfected for 30 h (A) and 48 h (B), followed by Hoechst staining. Images were taken using a 60× objective with a fluorescence microscope. DIC, bright-field microscopic image; DsRed1, red fluorescence; Merge, merger of images. Note the morphological changes seen in the form of rounding-off of the cells (30 h [A]) and cell death and disruption (48 h [B]). Download FIG S3, JPG file, 1.9 MB. Copyright © 2018 Grover et al. 2018 Grover et al. This content is distributed under the terms of the Creative Commons Attribution 4.0 International license.

### Disorder region analysis and ER localization signal sequence prediction.

GlobPlot analyses revealed comparatively few differences between the domains of Rv0297 and Rv1818c (Fig. S4). The main difference was the extended disorder region in the Rv0297/Rv0297PGRS domain compared to the Rv1818c/Rv1818cPGRS domain. The Rv0297PE domain alone did not show the presence of any disorder region or globular domain, possibly because of its short sequence, while the PE domain of Rv1818c was found to be globular.

10.1128/mBio.01017-18.4 FIG S4  Disorder analysis by the GlobPlot tool revealed few differences between the domains of Rv0297 and Rv1818c. The disorder has been depicted on the *x* axis in the form of an orange line. The extended disordered region in the Rv0297 protein/Rv0297 PGRS domain compared to the Rv1818c/Rv1818c PGRS domain is noticeable. Download FIG S4, TIF file, 2 MB. Copyright © 2018 Grover et al. 2018 Grover et al. This content is distributed under the terms of the Creative Commons Attribution 4.0 International license.

LocSigDB listed 29 variants of ER localization/retention sequences (Table S3), out of which 7 were found to be present in the Rv0297PGRS domain (highlighted in Table S3). Most of these putative ER-specific signals present in Rv0297 were similar to ER-residing proteins and viral proteins such as those in rubella virus and hepatitis virus. The presence of ER localization signals was interesting given the fact that another PGRS protein, Rv1818c, was known to be targeted to the mitochondria. This formed the basis for conducting *in vivo* ER localization studies on Rv0297 protein.

### Rv0297PGRS-PE combinations lead to ER localization.

**FIG 1:**
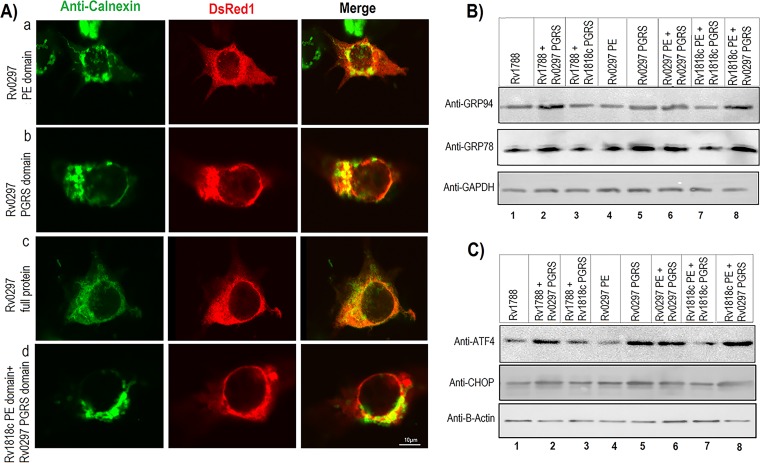
ER localization and activation of ER stress pathway by the PGRS domain of Rv0297. (A) Rv0297 colocalizes to the endoplasmic reticulum of transfected host cells. HEK293 cells were transfected with pDsRed1C1 vector containing the PE domain of Rv0297 (row a), the PGRS domain of Rv0297 (row b), the full-length Rv0297 protein carrying both PE and PGRS domains (row c), and the PE domain of Rv1818c fused with the PGRS domain of Rv0297 (row d). Cells with DsRed1 fluorescence (center column) were stained with ER-specific anticalnexin antibodies (left column), and merged images (right column) were created. Magnification, ×2,000. (B and C) PGRS domain of Rv0297 induces UPR pathway proteins and ER stress markers in transfected macrophage cells. Immunoblot analysis of murine macrophage RAW 264.7 cells with various constructs after 30 h of transfection. Total protein concentrations were determined, and equal amounts of proteins (25 µg/lane) were separated by 10% SDS-PAGE and blotted onto a polyvinylidene difluoride membrane followed by immunoblotting with anti-GRP78 and anti-GRP94 antibodies (B) or anti-ATF4 and anti-CHOP antibodies (C) as markers of the UPR pathway and ER stress, respectively. Glyceraldehyde-3-phosphate dehydrogenase (GAPDH) or β-actin was taken as a loading control. It can be seen that GRP78/GRP94 and ATF4/CHOP were overexpressed in cells transfected with the Rv0297 PGRS domain alone or in combination with other PE domains, compared to the PE control.

As PE_PGRS proteins are involved in several cellular functions, it was of interest to determine the localization of Rv0297PGRS in various subcellular locations. Rv1818c, a PGRS protein, is known to be localized to the mitochondria. Therefore, to validate the *in silico* data, experiments were performed to determine the effect of Rv0297PGRS on its subcellular localization. Colocalization studies were carried out using antibodies against calnexin, specific to the ER, and MitoTracker green for mitochondria. Results showed that in the case of Rv0297, expression of the full-length protein, its PGRS domain, or the PGRS domain fused with the PE domain of other proteins did not lead to mitochondrial localization (Fig. S5). However, for Rv1818c the full-length protein and its PGRS domain were found to localize to the mitochondria (Fig. S5) (18). However, coimmunostaining with calnexin antibody indicated ER localization for both Rv0297PGRS alone and its full-length protein (Fig. 1A). These results revealed that Rv0297PGRS appeared to target the ER in addition to other cellular organelles.

10.1128/mBio.01017-18.5 FIG S5  The Rv0297PGRS domain does not localize to the mitochondria of transfected host cells. HEK293 cells were transfected with the pDsRed1C1 vector containing the full-length Rv0297 protein carrying both the PE and PGRS domains (a) and the full-length Rv1818c protein carrying both the PE and PGRS domains (b). Cells with DsRed1 fluorescence (center panel) were stained with MitoTracker green (left panel), and merged images (right panel) were created. Magnification, ×1,000. Download FIG S5, TIF file, 0.8 MB. Copyright © 2018 Grover et al. 2018 Grover et al. This content is distributed under the terms of the Creative Commons Attribution 4.0 International license.

### The Rv0297PGRS domain is associated with upregulation of the UPR pathway within the ER.

Having shown the ability of the PGRS domain to mediate ER localization, we then considered the functional consequences of such targeting. To begin with, we determined the effects of the accumulation of the Rv0297PGRS domain alone or in combination with other PE domains on host cell functions. Given the association of the ER with the UPR, the levels of UPR pathway proteins, such as GRP78 and GRP94, were investigated. We simultaneously explored the levels of ATF4 (28) and CHOP (29), well-known ER stress markers. These transcription regulators target genes that are involved in host cell apoptosis (30). Immunoblotting studies clearly showed the overexpression of GRP78/GRP94 and ATF4/CHOP, compared to the PE control, in cells transfected with the Rv0297PGRS domain alone or in combination with other PE domains (Fig. 1B and C). Transfection with the Rv0297PGRS domain alone or the full-length protein in murine macrophages, as opposed to the PE domain, induced overexpression of GRP78 and GRP94 (Fig. 1B and C, compare lanes 5 and 6 with lane 4) as evident from immunoblotting. A construct carrying the Rv0297PGRS domain fused with Rv1788PE protein (Fig. 1B and C, lane 2) and the Rv0297PGRS domain fused with the Rv1818cPE domain (Fig. 1B and C, lane 8) was able to induce the overexpression of these ER-specific chaperones/stress markers compared to their respective native proteins. These results were also verified by densitometric analysis (Fig. S6). Having shown that the effect observed was not due to the generic PGRS domain but the specific PGRS domain sequence present in Rv0297, which activated the ER stress pathway, we then investigated the downstream consequences of ER stress.

10.1128/mBio.01017-18.6 FIG S6  Densitometry analysis of the Western blotting images developed with ER stress markers. Bar graphs obtained by densitometric analysis of Western blot data show the overexpression of ER stress markers GRP78 (a), GRP94 (b), ATF4 (c), and CHOP (d) in the lanes where the PGRS domain of Rv0297 was expressed. The values given were obtained with AlphaEaseFC software. The integrated density value (IDV) of each band was detected by drawing a rectangle outlining the band using the spotdenso tool of the software. Download FIG S6, TIF file, 0.2 MB. Copyright © 2018 Grover et al. 2018 Grover et al. This content is distributed under the terms of the Creative Commons Attribution 4.0 International license.

### Rv0297PGRS aids in intracellular Ca^2+^ release.

**FIG 2:**
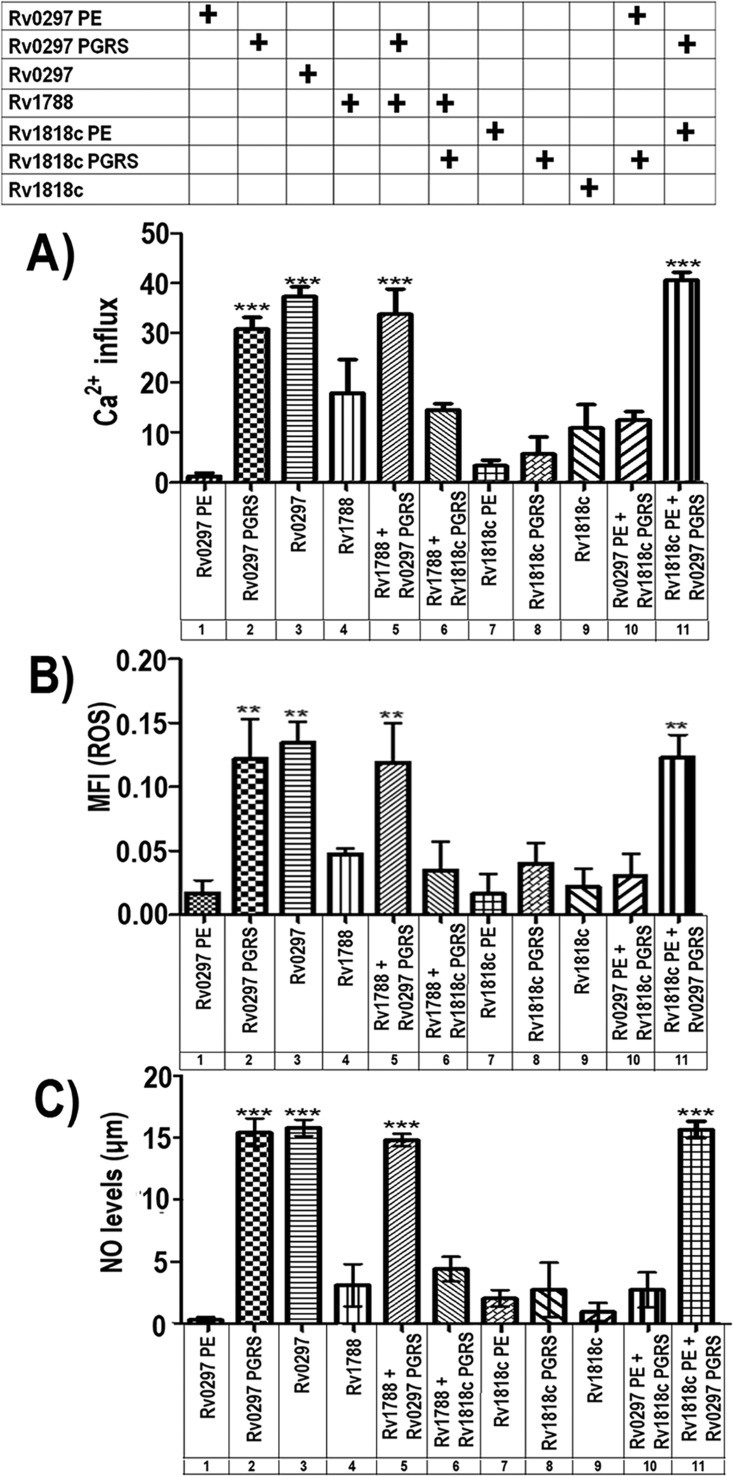
ER localization of the Rv0297PGRS domain disrupts Ca^2+^ homeostasis and increases ROS and NO levels. Ca^2+^, ROS, and NO levels were measured in RAW 264.7 cells after transfection with various plasmids carrying the constructs as indicated. Data shown for different experiments represent the mean and standard error from three independent assays performed in triplicate. The statistical comparisons were made by one-way ANOVA. *P* values for ** and *** are <0.01 and <0.001, respectively. (A) Disturbance of Ca^2+^ homeostasis by PGRS domain of Rv0297. Measurements of cytosolic Ca^2+^ responses were made using cells labeled with Fluo-4 NW dye for 30 min, and Ca^2+^ release was measured using a 96-well fluorescence plate reader with excitation at 494 nm and emission at 516 nm. A significant increase in Ca^2+^ after transfection with the constructs having the Rv0297PGRS domain was observed. (B) The PGRS domain of Rv0297 induces ER stress through ROS generation. Total reactive oxygen species (ROS) levels were measured using 5 µM CellROX green reagent for each well of the 96-well plate for 30 min at 37°C. Fluorescence was measured with an excitation wavelength of 485 nm and an emission wavelength of 520 nm. Note that cells transfected with constructs containing the Rv0297PGRS domain exhibited a 5- to 6-fold increase in the levels of ROS. (C) The PGRS domain of Rv0297 induces generation of nitric oxide. NO was quantitated using Griess reagent as described in Materials and Methods. The culture supernatants from RAW 264.7 cells transfected with various constructs were collected, and NO was measured. Relative NO concentrations were calculated by extrapolation of values from test samples on standard curves generated using sodium nitrite. A significant increase in the production of NO was observed (~15-fold) in RAW 264.7 cells transfected with constructs carrying the Rv0297PGRS domain.

The ER lumen is the key repository site of intracellular Ca^2+^ and Ca^2+^ binding chaperones which facilitate correct folding of native proteins (31). Changes in the levels of Ca^2+^ inside the ER lumen can substantially influence protein folding, leading to activation of cell death pathways. Extending the above results, we measured the levels of Ca^2+^ ion release from the ER, in cells transfected with various constructs. After 30 h of transfection of RAW 264.7 cells, there was a noticeable increase in Ca^2+^ after transfection with constructs carrying Rv0297PGRS (Fig. 2A, columns 2, 3, 5, and 11). Ca^2+^ efflux levels were not statistically significant (Fig. 2A, columns 6, 8, and 10) when constructs having the PGRS domain of Rv1818c were used. As variations in Ca^2+^ dynamics perform a significant role in the ER stress-mediated cell death mechanism, these results were supportive of the idea that Rv0297PGRS might be involved in host cell apoptosis.

### Rv0297PGRS domain-mediated Ca^2+^ release leads to ROS generation.

Perturbations in the intra-ER calcium species affect the production of ER stress-induced reactive oxygen species (ROS) (32–34). Numerous pieces of evidence support a linkage between generation of ROS and the UPR. Oxidative stress and ROS production are vital constituents of ER stress and are not just consequences of ER stress induction. We therefore determined the cellular ROS levels using CellROX green reagent. The results show that Rv0297PGRS alone (Fig. 2B, column 2) or in combination with other PE domains (Fig. 2B, columns 3, 5, and 11) greatly enhanced the cellular ROS levels in transfected macrophages. Compared to the PE domain of Rv0297 (column 1), PGRS domain-harboring cells (columns 2, 3, 5, and 11) showed a 5- to 6-fold increase in the levels of ROS. These results therefore established a link between Rv0297PGRS expression in macrophage cells and a role in endogenous ROS regulation.

### The Rv0297PGRS domain also causes generation of NO.

Increased levels of nitric oxide (NO) disturb ER calcium pump activity and also induce ER stress (35). We accordingly investigated the role of the PGRS domain present within Rv0297 in NO synthesis. As could be seen, RAW 264.7 cells transfected with a plasmid carrying the Rv0297PE domain alone secreted basal levels of NO (Fig. 2C, column 1), while production of NO was significantly increased ~15-fold in those expressing Rv0297PGRS alone or as a full-length protein or fused with Rv1788 PE protein or the Rv1818c PE domain (columns 2, 3, 5, and 11, respectively). These results further indicated an involvement of the Rv0297PGRS domain in activation of cellular NO species.

### Nuclear blebbing was observed in cells transfected with the Rv0297PGRS domain.

**FIG 3:**
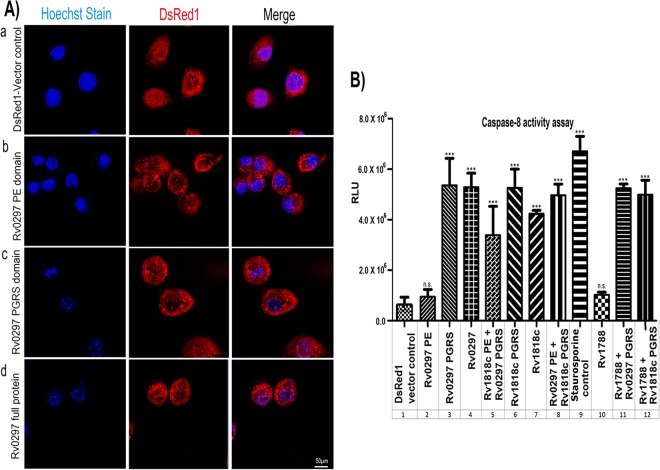
ER localization of the Rv0297PGRS domain induces apoptosis. (A) Nuclear blebbing was induced by the PGRS domain of Rv0297. HEK293 cells were transfected with DsRed1 vector control (row a), the PE domain of Rv0297 (row b), the PGRS domain of Rv0297 (row c), and full-length Rv0297 protein carrying both PE and PGRS domains (row d). Cells transfected with these constructs were fixed and stained with Hoechst nuclear stain and observed under a fluorescence microscope using a 100× objective. Cells with DsRed1 fluorescence (center column) were stained with Hoechst nuclear stain (left column), and merged images (right column) were created. (B) Activation of caspase-8-mediated apoptosis. Caspase-8 activity was measured using the Caspase-Glo assay kit (Promega). Staurosporine was used as a positive control for apoptosis. Data shown represent the mean and standard error from three experiments performed in duplicate. The statistical comparisons were made by one-way ANOVA. *P* values for *** and n.s. are <0.001 and >0.05, respectively. A significantly elevated 5- to 6-fold increase in the levels of caspase-8 could be seen in cells transfected with constructs carrying the PGRS domain of Rv0297 compared to those transfected with the PE domains.

Macrophage cells were transfected with various constructs, including the PE domain of Rv0297 (Fig. 3A, row b), Rv0297PGRS (row c), or full-length Rv0297 protein (row d), along with vector control (row a) for 30 h. DsRed1 signals when merged with Hoechst nuclear stain highlighted the relative intensity of blebbing (right column). Analyses of fluorescence microscopic images revealed that wherever Rv0297PGRS was present, a clear nuclear blebbing was observed (Fig. 3A). These results clearly pointed to the role of the PGRS domain of Rv0297 in the induction of apoptosis in macrophage cell lines.

### Caspase-8 is activated by the Rv0297PGRS domain.

To confirm the results described above, activation of caspase-8, the effector caspase of the cellular apoptotic pathway, was assayed. Caspase-8 activity was measured in RAW 264.7 cells transfected with the Rv0297PGRS construct. A significantly elevated (5- to 6-fold) increase in the levels of caspase-8 could be seen in RAW 264.7 cells transfected with constructs carrying Rv0297PGRS alone (Fig. 3B, column 3) or full-length Rv0297 (column 4) or Rv0297 fused with the PE domain of Rv1818c (column 5) or Rv1788 PE protein (column 11). The activation of caspase-8 noticed for constructs carrying the PGRS domain of Rv1818c (column 6) or full-length Rv1818c (column 7) or Rv1818c fused to the PE domain of Rv0297 (column 8) or Rv1788 PE protein (column 12) was expected since the Rv1818c PGRS domain is known to cause apoptosis involving the mitochondrial apoptotic pathway. Interestingly, the PE domain alone of Rv0297 (column 2) or Rv1788 PE protein (column 10) was unable to cause caspase-8 activation. These results demonstrated that induction of apoptosis by Rv0297PGRS is facilitated by classical caspase-8-dependent pathways, similarly to Rv1818c-mediated apoptosis (36).

### *In vivo* localization of Rv0297 from *M. tuberculosis* H_37_Rv to the ER of RAW 264.7 cells.

**FIG 4:**
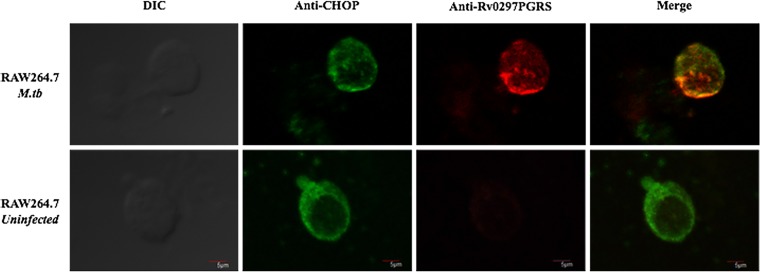
*In vivo* localization of Rv0297 from H_37_Rv to the ER in *M. tuberculosis*-infected RAW 264.7 macrophages. *M. tuberculosis*-infected RAW 264.7 macrophages were stained with anti-CHOP antibody ER-specific marker (second column) and anti-Rv0297PGRS sera (third column), and merged images (last column) were created. Colocalization of Rv0297 to the ER of infected macrophages was found. Magnification, ×1,200. DIC, differential inference contrast.

Colocalization of PE_PGRS5 to the ER in pDsRed1C1-Rv0297-transfected cells was observed in previous results. To validate this, *in vivo* colocalization of Rv0297 to the ER of infected macrophages was performed. Uninfected RAW 264.7 cells were used as a negative control. Confocal microscopy analysis revealed the colocalization of *M. tuberculosis* Rv0297 into the ER of infected macrophages (Fig. 4). Merged images clearly indicated the presence of Rv0297 in the ER of infected macrophages (Fig. 4, upper panels) but not in uninfected ones (Fig. 4, lower panels). These observations corroborate our previous results where overexpression of Rv0297 was done in HEK293T cells.

### Rv0297PGRS protein induces ROS and NO production and host cell apoptosis.

**FIG 5:**
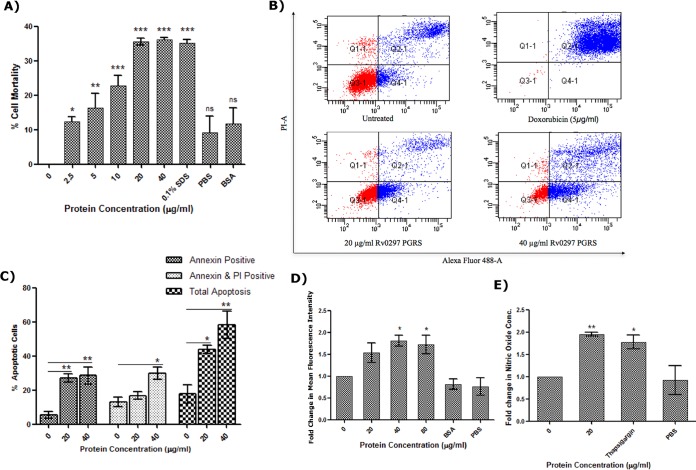
Rv0297PGRS protein induces ROS and NO production and host cell apoptosis. (A) Induction of RAW 264.7 cell death by the recombinant Rv0297PGRS protein of *M. tuberculosis*. RAW 264.7 cells were incubated with different concentrations of recombinant Rv0297PGRS for 48 h, and cell mortality was assessed by alamarBlue assay. SDS (0.1%) was used as a positive control; 50 µg/ml of BSA was used as a negative control. (B) Apoptosis of RAW 264.7 cells observed upon stimulation with 20 and 40 µg/ml of recombinant Rv0297PGRS protein. Untreated, negative control; doxorubicin, positive control for induction of apoptosis; Q1-1 (annexin V negative, PI positive), dead cells; Q2-1 (double positive), late apoptosis; Q3-1 (double negative), live cells; Q4-1 (annexin V positive, PI negative), early apoptosis. (C) Percentage of apoptotic cells. Group I, early apoptotic cells; group II, late apoptotic cells; group III, total apoptotic cells (sum of early and late apoptotic cells) as calculated from groups I and II. (D) ROS generation by macrophage cells upon stimulation with recombinant Rv0297PGRS for 30 h. Eighty micrograms per milliliter of BSA was used as a negative control. Data were plotted as fold change of mean fluorescence intensity of CellROX reagent. (E) NO production by macrophage cells upon stimulation with recombinant PGRS domain of Rv0297 for 30 h. One micromolar thapsigargin was used as a positive control. Data were plotted as fold change in nitric oxide concentration. All values were represented as means ± SDs from three independent experiments. *P* values for *, **, ***, and ns are <0.05, <0.01, <0.001, and >0.05, respectively.

Consistent with transfection results, the recombinant protein also efficiently induced cell death of RAW 264.7 macrophages. The viability of cells was significantly decreased in the presence of rRv0297PGRS as evident from alamarBlue assay (Fig. 5A). rRv0297PGRS protein (2.5 µg/ml) induced 12.42% ± 1.26% mortality of macrophage cells, and this increased as a direct function of the protein concentration: 16.24% ± 4.29%, 22.79% ± 2.90%, 35.48% ± 0.98%, and 36.07% ± 0.61% with 5, 10, 20, and 40 µg/ml rRv0297PGRS, respectively. These results demonstrate that administration of the rRv0297PGRS protein to macrophages leads to cell death in a dose-dependent manner.

In order to determine the mode of cell death, annexin V/propidium iodide (PI) staining of rRv0297PGRS-treated RAW 264.7 cells was used. Both early and late apoptosis of cells were observed upon stimulation with 20 and 40 µg/ml of rRv0297PGRS protein (Fig. 5B). The total percentage of apoptotic cells induced by 20 and 40 µg/ml of rRv0297PGRS was found to be 43.83% ± 2.54% and 58.37% ± 7.92%, respectively (Fig. 5C), indicating that Rv0297PGRS is inducing apoptosis rather than necrosis.

The effect of rRv0297PGRS protein on generation of ROS and NO and their involvement in ER stress was also assessed. Increased production of ROS and NO macrophages was observed upon stimulation with rRv0297PGRS protein. Fold increments of 1.53 ± 0.22 and 1.80 ± 0.13 in the levels of ROS were observed compared to untreated cells (Fig. 5D). Almost a 2-fold increase in the levels of NO was observed in cells stimulated with 20 µg/ml of rRv0297PGRS protein in contrast to unstimulated cells (Fig. 5E). Bovine serum albumin (BSA) (80 µg/ml) induced negligible amounts of ROS and NO. These results support the role of Rv0297PGRS in ER stress-mediated apoptosis.

### *In silico* analysis revealed preferential binding of Rv0297 with TLR4 compared to TLR2.

**FIG 6:**
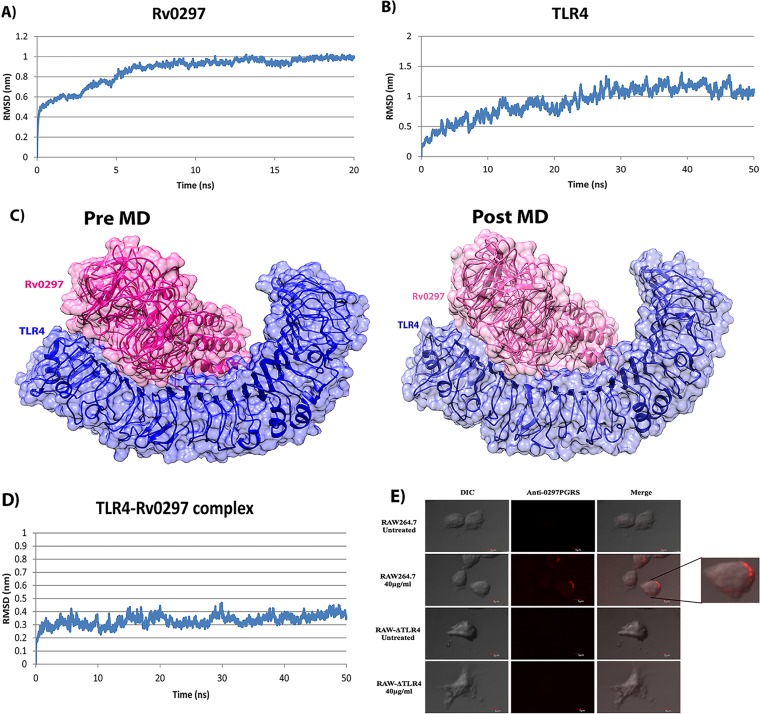
Interaction of Rv0297 and TLR4. (A to D) *In silico* interaction analysis of Rv0297 and TLR4. (A) Backbone RMSD of Rv0297 during 20-ns MD simulation. (B) Backbone RMSD of TLR4 during 50-ns MD simulation. (C) Pre-MD and post-MD surface interaction diagram of TLR4-Rv0297 complex. (D) Backbone RMSD of TLR4-Rv0297 complex during 50-ns MD simulation. (E) *In vitro* interaction of TLR4 with the PGRS domain of Rv0297 protein of *M. tuberculosis*. RAW 264.7 and RAW-ΔTLR4 cells incubated with recombinant Rv0297PGRS protein for 2 h and stained with anti-Rv0297PGRS sera. Unstimulated cells were used as a negative control. Alexa Fluor 555 fluorescent images were captured for TLR4 and Rv0297PGRS interaction (middle column), and merged images were created (right column). Magnification, ×1,200.

Host-pathogen cross talks mostly involve pathogen recognition receptors (PRRs) or Toll-like receptors (TLRs) of host and pathogen-associated molecular patterns (PAMPs) of microbes. Few of the PE_PGRS proteins of *M. tuberculosis* were known to interact with TLRs, mainly the TLR2 type. We therefore carried out *in silico* simulations to predict the identity of the TLR involved in interaction. The I-TASSER-predicted model was chosen on the basis of the high confidence score (C-score), 0.26, and the PSVS results. Verify 3D (reference range, −1 to +1) also confirmed the good model quality with a score of 0.56. The modeled three-dimensional (3D) structure was refined through molecular dynamics (MD) simulations and showed a stable trajectory from 6.5 to 20 ns with small root mean square deviations (RMSDs) between 0.8 nm and 1 nm (Fig. 6A). The model quality significantly improved with a Verify 3D score of 0.58 and overall 98.8% residues in the favored or allowed region in comparison to 97.7% before MD. Only 1.2% amino acids were in the disallowed region (earlier value, 2.4%).

MD refinement of the TLR4 structure also shows a stable trajectory from 30 ns to 50 ns (Fig. 6B). Protein-protein docking was carried out using HADDOCK 2.2 for comparative analysis of Rv0297 interaction with TLR4 versus TLR2. We obtained a −13.9 ± 14.5 HADDOCK score for TLR4 in comparison to −5.3 ± 17.8 for TLR2. HADDOCK clustered 106 structures into 11 clusters for the TLR4-Rv0297 complex, while it clustered 41 structures into 7 clusters for the TLR2-Rv0297 complex. The TLR4-Rv0297 complex structure was taken from cluster 2 on the basis of the best Z score, −2.2, while the TLR2-Rv0297 complex from the best cluster, 1, had a Z score of −1.6. These *in silico* studies suggested that TLR4 may have a better interaction with Rv0297 than does TLR2. The TLR4-Rv0297 complex was then analyzed for stable interaction after long-term MD simulations. The backbone RMSD of the TLR4-Rv0297 complex shows a stable trajectory of 50 ns (Fig. 6D). Comparison of pre-MD and post-MD complexes by LigPlot+ (37) revealed conserved interactions in the form of hydrogen or hydrophobic bonds (Table S4). The surface interaction diagram of the TLR4-Rv0297 complex also disclosed a perfectly fitting interaction between TLR4 and Rv0297 (Fig. 6C).

### *In vitro* interaction assay of TLR4 and PGRS domain of Rv0297.

Having predicted TLR4 interaction with Rv0279PGRS, we demonstrated the same by *in vitro* interaction assay. RAW 264.7 and RAW-ΔTLR4 cells were incubated with rRv0297PGRS protein and immunostained with anti-Rv0297PGRS sera to assess the interaction of TLR4 localized on the surface of macrophages with Rv0297PGRS protein. Confocal imaging revealed the presence of the interacting complex on the surface of RAW 264.7 cells incubated with rRv0297PGRS protein (Fig. 6E, row 2). No such interaction was observed without any protein (Fig. 6E, row 1). In contrast to RAW 264.7 cells, RAW-ΔTLR4 cells did not display such complexes on their surface even in the presence of rRv0297PGRS protein (Fig. 6E, row 4). The presence of this interaction on the surface of RAW 264.7 cells only and not on TLR4-deficient cells indicates that Rv0297PGRS indeed interacts with TLR4 of macrophages.

### The downstream effect of action of the PGRS domain is TLR4 dependent.

**FIG 7:**
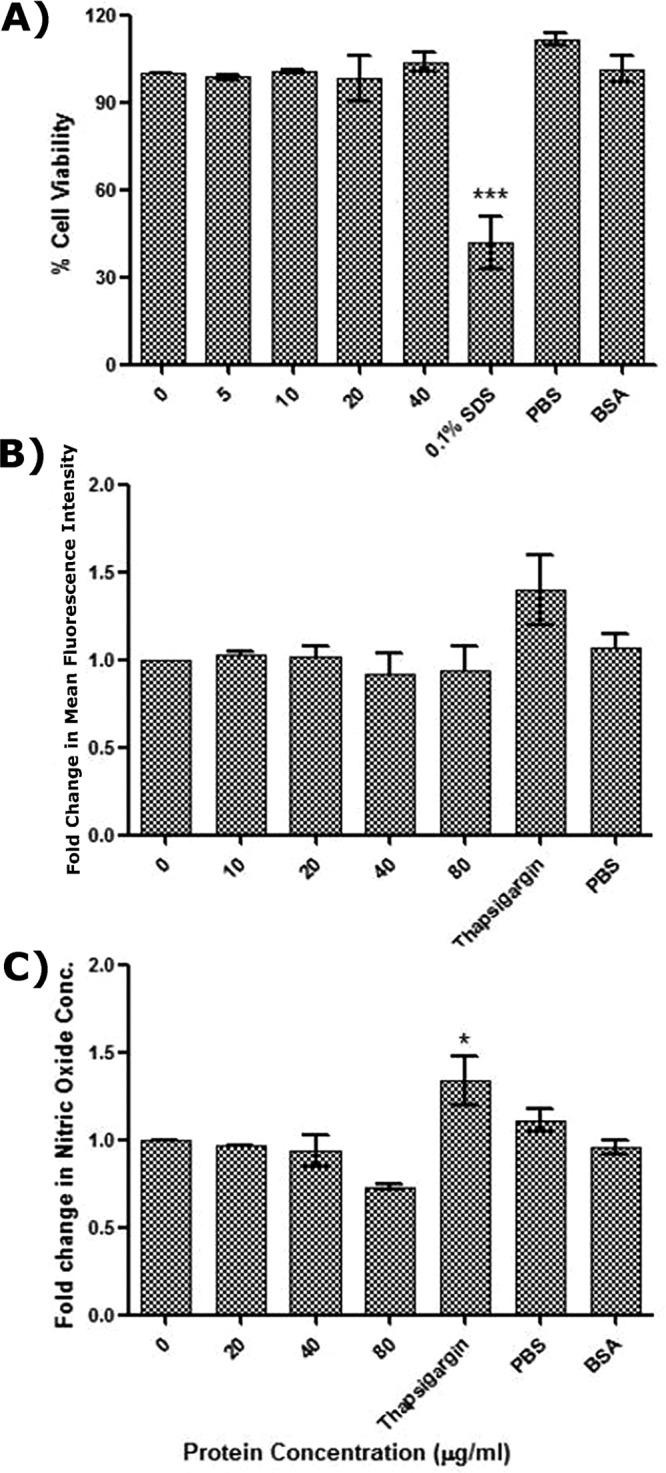
The effect of the PGRS domain of Rv0297 is TLR4 dependent. (A) Cell viability assessment of RAW-ΔTLR4 macrophages after 48 h of stimulation with recombinant Rv0297PGRS protein. SDS (0.1%) was used as a positive control; 50 µg/ml of BSA was used as a negative control. (B and C) Assessment of ROS (B) and NO (C) generation by RAW-ΔTLR4 macrophages upon stimulation with Rv0297PGRS. Data were plotted as fold change of mean fluorescence intensity (A) and as fold change of nitric oxide concentration (B). Eighty micrograms per milliliter of BSA was used as a negative control. One micromole thapsigargin was used as a positive inducer for ER stress. All values were represented as means ± SDs from three independent experiments.

RAW-ΔTLR4 cells were stimulated with 0 to 40 µg/ml of rRv0297PGRS protein for 48 h, and the cell survival was assessed by alamarBlue assay. No significant change in cell survival was observed upon rRv0297PGRS protein treatment in comparison to untreated cells and 50-µg/ml BSA control (Fig. 7A). All cells were healthy and alive even upon 40-µg/ml rRv0297PGRS protein stimulation. On the other hand, decreased viability was observed in the case of 0.1% SDS treatment (positive control). The results clearly indicate the role of TLR4 in the effect of Rv0297PGRS protein. The levels of ROS and NO were then assessed once again to further validate the role of TLR4 in rRv0297PGRS protein-stimulated ER stress-mediated apoptosis. To investigate this, RAW-ΔTLR4 cells were treated with rRv0297PGRS (0 to 40 µg/ml) and the levels of ROS and NO were assayed and found to be exactly similar in both treated and untreated cells (Fig. 7B and C, respectively). In contrast to this, 1 µM thapsigargin (positive control) efficiently induced almost 50% increase in both ROS and NO levels from RAW-ΔTLR4 cells. These results demonstrate that the downstream effects of Rv0297PGRS protein, such as cell death and ROS and NO production, are TLR4 dependent.

## DISCUSSION

Intracellular pathogens often act to prevent host cell apoptosis to reduce the immune response. Previous studies revealed that apoptosis is a macrophage defense process to counter *M. tuberculosis* infection (38). Despite the fact that *M. tuberculosis* is able to inhibit apoptosis in macrophages, it is still capable of inducing apoptosis (39). Apoptotic macrophages have been found within *M. tuberculosis*-infected lung granulomas (40, 41). These findings generate considerable interest in the potential role of apoptosis in *M. tuberculosis* infection and subsequent pathogenesis.

Previous studies also emphasized that ER stress-mediated apoptosis plays an important role in TB pathogenesis (22, 23). ER stress has been found to induce apoptosis in TB granulomas, in zones where apoptotic macrophages accumulate in mice and humans (21). The appearance of Rv0297 protein in TB granuloma 90 days postinfection and its absence during initial phases of infection (30 days postinfection) point to its role in granuloma maintenance (24). However, the exact mechanism by which it helps in *M. tuberculosis* survival in the human host and maintenance of disease is not known.

Using various fusion constructs and colocalization studies, we showed that Rv0297 protein localizes to the ER, independent of its PE domain. In order to decipher the biological effects of localization within the ER of the host cells, we investigated the ER stress pathways in cells harboring various domains of Rv0297 as well as another PE protein (Rv1788) and PE_PGRS protein (Rv1818c). Our results show that the Rv0297 PGRS domain is involved in ER stress. Notably, it has been shown that when stress is excessively high, the UPR pathway shifts from a prosurvival to a prodeath mode (42). GRP78 and GRP94 are well-established ER chaperone molecules which have a crucial role in maintaining cell viability under numerous cell stresses (43). Under pathological conditions, altered levels of ATF4 and CHOP—crucial proteins expressed during ER stress-induced apoptosis—have been shown (22). Using GRP94, GRP78, ATF4, and CHOP as standard UPR markers for ER stress, we showed upregulation of these markers as a consequence of Rv0297PGRS domain expression in macrophages. Together, these data suggest that, in later stages of *M. tuberculosis* infection, the ER stress sensors might be upregulated for induction of apoptosis as a reaction to *M. tuberculosis* infection. Disruption of Ca^2+^ homeostasis is a contributing element of ER stress. We therefore also investigated the possible involvement of the Rv0297PGRS-mediated Ca^2+^ ion release in ER stress in RAW 264.7 cells. The levels of intracellular Ca^2+^ ions in RAW 264.7 cells were shown to be elevated using a fluorescent Ca^2+^ indicator. ROS and NO generation represent essential mechanisms invoked by macrophages to control *M. tuberculosis* (44). These antimicrobial species play a critical role in controlling intracellular bacteria. Interestingly, ER stress pathways are also induced by ROS or NO (45). Our results indicate that Rv0297PGRS induces both NO and ROS production in RAW 264.7 cells. We also showed that ectopic expression of the PGRS domain affects the levels of caspase-8, an effector caspase usually activated by the extrinsic apoptotic pathway (46). Caspase-8 and caspase-3 are the key caspases involved in ER stress-mediated apoptosis (47).

Pathogens use intrinsically disordered proteins to perturb and hijack host cell networks for a productive infection (48, 49). An increased disordered region present in Rv0297PGRS might be responsible for differences in organelle localization and function from Rv1818cPGRS. Fusion of Rv0297PGRS with other PE domains allowed them to be targeted to the ER. Protein sequence alignment revealed some extra sequences in the Rv0297PGRS domain, possibly containing motifs responsible for ER localization. Rv1818c, a well-characterized protein known to be localized to mitochondria, while sharing some common motifs apparently lacks a few of them (see Table S3 in the supplemental material). While some ER signal sequences are present in Rv0297PGRS, we were unable to identify the precise signature motifs which enable exclusive targeting to the ER. It is presumable that these sequences might not be responsible for organelle-specific targeting but could be responsible for organelle targeting generically. Furthermore, some of these signals might be responsible for retention of Rv0297PGRS in the host ER, leading to generation of the stress response and induction of caspase-8-mediated apoptosis.

Very little is known about the functional role of PE_PGRS proteins, as only a few PE_PGRS proteins have been assigned a physiological function so far (3). For example, the PE domain Rv1818c, a well-studied protein of this family, induces primary necrosis, whereas the PGRS domain is involved in induction of mitochondrion-mediated apoptosis. Recently, it was demonstrated that PE_PGRS30 is required for virulence, as its deletion from *M. tuberculosis* compromised its capacity to colonize in lung tissue and hampered tissue damage. The inactivation of the gene also leads to inhibition of phagolysosome fusion in macrophages (50). Similarly, *M. tuberculosis* PE_PGRS17 promotes the death of host macrophages as well as increases in the secretion of the proinflammatory cytokine tumor necrosis factor alpha (TNF-α) (51). On the other hand, PE_PGRS62 reduces phagolysosome maturation and induces secretion of gamma interferon (IFN-γ) (52).

Induction of ER stress-mediated cell death via apoptosis has been described in several diseases, including vascular diseases (53). The ER stress pathway was initially recognized as a cellular mechanism activated by the accumulation of unfolded proteins in the ER to maintain proper ER functions. In addition, ER stress pathways are also involved in protection of cells by several other cellular stresses. However, when stress reaches a critical level, apoptosis is induced to eliminate injured cells (54). In later stages of *M. tuberculosis* infection, the consequence of cell death favors the pathogen. A mature TB granuloma in human patients has slight vascularization and a limited access to immune cells such as macrophages and lymphocytes (25). The stimulation of cellular apoptosis in the infected and foamy macrophages, which are profusely present, will cause accumulation of caseum and the development of disease pathology. *M. tuberculosis* takes benefit of this pathology by liquefaction and cavitation of the granuloma, in order to transmit infectious bacilli into the airways. As a result, whereas apoptosis could be advantageous to the host at low bacterial numbers at early stages of infection, it is harmful after the disease has advanced and fibrocaseous granulomas have been generated. Mycobacterial phenolic glycolipid-dependent production of CCL2 in human alveolar macrophages serves as a source for recruitment of permissive macrophages to sustain and disseminate the mycobacterial infection (55).

The earliest step in an infection is the interaction of host cell and microbial components. Macrophages sense or recognize specific conserved moieties of microbes called PAMPs with the help of PRRs expressed on their surface. Interaction of TLRs of macrophages with different *M. tuberculosis* ligands is required for the modulation of various cellular events such as apoptosis of macrophages (56), production of ROS and NO intermediates (57), antigen presentation (58), and phagolysosomal fusion (59).

Pathogenesis and virulence of *M. tuberculosis* depend on TLR1, TLR2, TLR4, and TLR9 and their signaling cascades (60). Induction of host cell apoptosis through TLR2 signaling has been shown by stimulation with mycobacterial ligands and live *M. tuberculosis*. Few of the PE/PPE proteins such as Rv1818c of *M. tuberculosis* have been shown to stimulate host cell apoptosis in a TLR2-dependent manner (36). Maturation and activation of dendritic cells by PE_PGRS11 and PE_PGRS17 were shown to be dependent on TLR2 signaling (61). However, it is yet to be established whether TLR2 interaction is the common property of PE/PPE proteins. The complex interactive network of PE/PPE proteins with host cell receptors modulates the pathogenesis of disease. In contrast, an important role of both TLR2 and TLR4 in induction of macrophage apoptosis upon *M. tuberculosis* infection has also been shown. TLR4-dependent signaling is important in maintaining the balance between necrosis and apoptosis of macrophages (62). We report for the first time the interaction of TLR4 with Rv0297PGRS of *M. tuberculosis* in execution of host cell apoptosis. *In silico* analysis revealed that Rv0297 interacts with TLR4, which was also corroborated by our *in vitro* TLR4 and Rv0297PGRS interaction assay. Confocal images of this interaction clearly indicated the presence of interacting complexes on the surface of RAW 264.7 cells, but they were totally absent on the cells lacking TLR4. Also, the induction of ROS and NO from macrophages was found to be dependent on TLR4 interaction. RAW cells lacking TLR4 were unable to undergo apoptosis upon stimulation with rRv0297PGRS. All these results supported the functioning of Rv0297 in a TLR4-dependent manner. Another mycobacterial PE protein complex, PE9/PE10, has also been shown to induce apoptosis of macrophages via its interaction with TLR4 (63).

**FIG 8:**
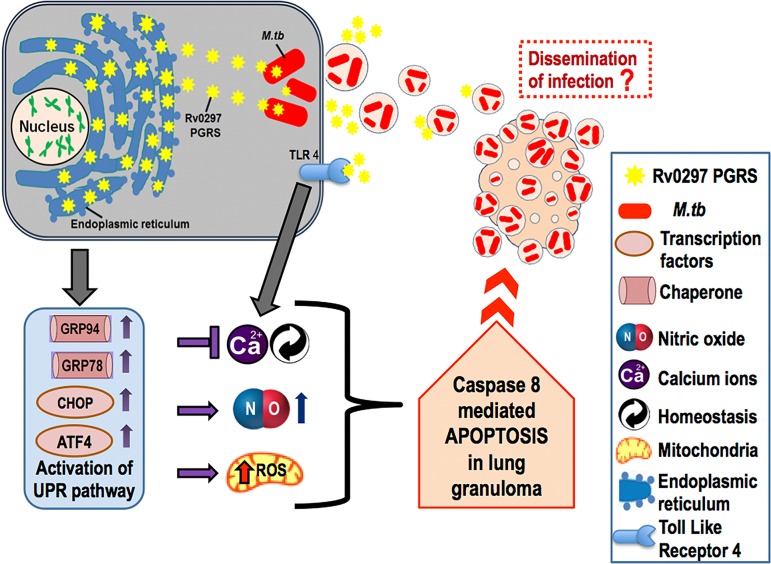
Proposed model of mode of action of *M. tuberculosis* Rv0297PGRS domain. Expression of the PGRS domain of Rv0297 in human cells leads to its ER localization, which results in the expression of proteins involved in the unfolded protein response (UPR) pathway, i.e., GRP94, GRP78, and ER stress markers, i.e., CHOP and ATF4. Induction of ER stress is followed by disruption of intracellular Ca^2+^ homeostasis and increased NO and ROS production. These ER stress responses result in apoptosis of macrophage cells, which may lead to dissemination of infection from lung granulomas.

The involvement of the PE_PGRS protein Rv0297 in ER stress-mediated induction of apoptosis in macrophages points to a novel role for the PGRS domain of this family of proteins. We propose that Rv0297 expression in human cells leads to its ER localization, followed by disruption of intracellular Ca^2+^ levels and induction of ROS and NO production (Fig. 8). The ensuing ER stress response culminates in caspase-8 activation and induces apoptosis in macrophage cells. Apoptosis of infected macrophages in advanced stages of granulomas would support dissemination of the bacteria through apoptotic bodies, thereby playing an important role in spreading the disease. The molecular dissection of the different domains within the PE_PGRS protein of the PE/PPE family, present exclusively in the genus *Mycobacterium*, and their possible functional significance will aid in a better understanding of the virulence and pathogenesis of *M. tuberculosis*. Future experiments with lung granulomas will likely provide a better insight into the mode of action.

## MATERIALS AND METHODS

### *In silico* analyses of Rv0297.

The GlobPlot 2.3 (http://globplot.embl.de/cgiDict.py) tool was used to predict disordered regions and globularity in the PE_PGRS proteins used in this study. The presence of putative ER localization signals in Rv0297 protein was searched from LocSigDB (64) using the FIMO tool (65) from MEME Suite version 4.11.2 (http://meme-suite.org/tools/fimo) with a *P* value of <0.001. The sequence file of the amino acid sequence of Rv0297 was uploaded under “Input the sequences,” and ER signal sequences were typed under “Input the motifs” in the data submission form.

### Protein structure modeling, validation, and protein-protein docking.

The Rv0297 protein sequence was obtained from the UniProtKB database (identifier [ID] Q6MX50) (66), and the 3D structure was modeled using I-TASSER (67). The human TLR4 protein structure (27 to 627 amino acids [aa]) was obtained from the RCSB protein data bank (PDB ID 3FXI). Rv0297 interactions with TLR4 and TLR2 were compared through the HADDOCK 2.2 web server (68). Structure validation was done through PSVS (69), and molecular dynamics (MD) simulations were performed with GROMACS v5.0.7 (70–74).

### Generation of constructs.

Genomic DNA of H_37_Ra was provided by Astrid Lewin, RKI, Berlin, Germany. The PE18 gene (Rv1788), Rv0297, Rv1818c full-length gene, and their PE and PGRS domains were cloned as C-terminal fusions into pDsRed1C1 vector. The primer sequences and PCR conditions are listed in Tables S1 and S2 in the supplemental material, respectively. A complete list and description of the constructs prepared are shown in Fig. S1.

10.1128/mBio.01017-18.7 TABLE S1  Primer sequences. Download TABLE S1, DOCX file, 0.1 MB. Copyright © 2018 Grover et al. 2018 Grover et al. This content is distributed under the terms of the Creative Commons Attribution 4.0 International license.

10.1128/mBio.01017-18.8 TABLE S2  PCR conditions for the amplicons. Download TABLE S2, DOCX file, 0.1 MB. Copyright © 2018 Grover et al. 2018 Grover et al. This content is distributed under the terms of the Creative Commons Attribution 4.0 International license.

For generating rRv0297PGRS protein, the Rv0297PGRS gene was cloned in the pET28a expression vector and expressed in BL21(DE3)pLysS cells. Recombinant protein was purified from inclusion bodies by solubilization in 8 M urea in phosphate-buffered saline (PBS) (pH 7.5) and on-column renaturation using a urea gradient followed by Ni^2+^-nitrilotriacetic acid (NTA) chromatography. The protein was treated with polymyxin B at 4°C for 2 h.

### Immunization and antibody generation.

A rabbit was immunized with 500 µg/ml of rRv0297PGRS, 3 booster doses of immunization were given at an interval of 15 days, and sera were collected 15 days after the last immunization.

### Cell culture and transfection.

The murine macrophage cell lines RAW 264.7 and RAW-ΔTLR4 and HEK293T (human embryonic kidney cells) were maintained in Dulbecco’s modified Eagle’s medium (Invitrogen) supplemented with 10% fetal bovine serum (FBS) (Invitrogen) and penicillin-streptomycin (Pen-Strep) (100 µg/ml). Cells (5,000/well) were seeded in a 96-well plate. The constructs were transfected into HEK293T cells for localization studies and in the RAW 264.7 cell line for ER stress-related experiments using Lipofectamine 3000 (Invitrogen) for 30 h and 48 h.

### Immunofluorescence staining.

HEK293T, RAW 264.7, and RAW-ΔTLR4 cells were seeded on coverslips to adhere for 16 to 18 h and transfected with constructs shown in Fig. S1 or stimulated with different concentrations of rRv0297PGRS. After the required period of incubation, cells were fixed with 3.7% paraformaldehyde for 15 min, permeabilized, and blocked with 0.1% Triton X-100–1% BSA–PBS for 10 min. The cells were incubated with the respective primary and Alexa Fluor-conjugated secondary antibodies (75). Images were visualized using an Olympus FluoView FV1200 laser scanning confocal microscope.

### *In vitro* infection of RAW 267.4 cells with *M. tuberculosis* H_37_Rv.

RAW 264.7 cells (2 × 10^6^ cells/well) were seeded and the next day infected with *M. tuberculosis* H_37_Rv (optical density [OD] at 600 nm, 0.8; multiplicity of infection [MOI], 1:10). After 3 h of infection, cells were washed with PBS and incubated with complete medium for 24 h.

### Cell death assay.

RAW 264.7 and RAW-ΔTLR4 cells were treated with rRv0297PGRS (2.5 to 40 µg/ml) for 48 h. Ten percent resazurin sodium salt (100 mg/ml) was added. Absorbance was monitored at 570 nm with reference at 600 nm after 24 h of reaction.

For annexin V/PI staining, treated cells (24 h) were stained with the Alexa Fluor 488 annexin V and PI flow cytometry kit (Invitrogen). Analysis of 10,000 stained cells was performed with a FACSAria 3 cytometer (BD Biosciences, USA).

### Calcium influx assay.

Transfected cells were stained with Fluo-4 NW dye solution (Molecular Probes) at 37°C for 30 min and further incubated at room temperature (RT) for 30 min. Calcium release was detected by measuring the fluorescence intensity using a 494-nm excitation and a 516-nm emission wavelength.

### Detection of ROS.

Transfected or treated cells were stained with 5 mM CellROX green reagent for 30 min at 37°C. Plates were read using a 485-nm excitation and a 520-nm emission wavelength. Results were represented as the mean fluorescent intensity of cells after subtraction of the blank (CellROX green reagent-unstained cells).

### NO quantitation in macrophages.

Cells were either transfected with various combination of PE and PGRS gene constructs or treated with 20 to 80 µg/ml of rRv0297PGRS protein for 30 h. Cell-free supernatant (150 µl) was mixed with 50 µl of Griess reagent for 30 min. The nitrite concentration was measured using sodium nitrite as a standard. Plates were read at 540 nm.

### Western blot analysis.

Western blotting was performed with anti-CHOP, anti-ATF4, anti-GRP94, and anti-GRP78/BiP antibodies (Abcam, USA) and anti-β-actin (Santa Cruz Biotechnology, USA). Membranes were developed with a chemiluminescent reagent (Millipore).

### Caspase assay.

The activity level of initiator caspase-8 was determined using the Caspase-Glo assay (Promega).

### Statistical analysis.

All data were expressed in the form of means ± standard deviations (SDs) derived from 3 different groups of independent experiments using GraphPad Prism5 software. A one-way analysis of variation (ANOVA) was performed followed by Dunnett’s *post hoc* test in order to calculate the statistical significance at a *P* value of <0.05.

10.1128/mBio.01017-18.9 TABLE S3  ER localization signal sequence analysis. Download TABLE S3, XLSX file, 0.03 MB. Copyright © 2018 Grover et al. 2018 Grover et al. This content is distributed under the terms of the Creative Commons Attribution 4.0 International license.

10.1128/mBio.01017-18.10 TABLE S4  Comparison of pre-MD and post-MD interactions of TLR4-Rv0297 complex. Download TABLE S4, DOCX file, 0.1 MB. Copyright © 2018 Grover et al. 2018 Grover et al. This content is distributed under the terms of the Creative Commons Attribution 4.0 International license.

## References

[1] World Health Organization 2015 Global tuberculosis report 2015, 20th ed. World Health Organization, Geneva, Switzerland.

[2] Sullivan T, Ben Amor Y 2012 The co-management of tuberculosis and diabetes: challenges and opportunities in the developing world. PLoS Med 9:e1001269. doi:10.1371/journal.pmed.1001269. 22911359 PMC3404121

[3] Akhter Y, Ehebauer MT, Mukhopadhyay S, Hasnain SE 2012 The PE/PPE multigene family codes for virulence factors and is a possible source of mycobacterial antigenic variation: perhaps more? Biochimie 94:110–116. doi:10.1016/j.biochi.2011.09.026. 22005451

[4] Mohareer K, Tundup S, Hasnain SE 2011 Transcriptional regulation of Mycobacterium tuberculosis PE/PPE genes: a molecular switch to virulence? J Mol Microbiol Biotechnol 21:97–109. doi:10.1159/000329489. 22286037

[5] Tundup S, Akhter Y, Thiagarajan D, Hasnain SE 2006 Clusters of PE and PPE genes of Mycobacterium tuberculosis are organized in operons: evidence that PE Rv2431c is co-transcribed with PPE Rv2430c and their gene products interact with each other. FEBS Lett 580:1285–1293. doi:10.1016/j.febslet.2006.01.042. 16458305

[6] Ahmad J, Farhana A, Pancsa R, Arora SK, Srinivasan A, Tyagi AK, Babu MM, Ehtesham NZ, Hasnain SE 2018 Contrasting function of structured N-terminal and unstructured C-terminal segments of Mycobacterium tuberculosis PPE37 protein. mBio 9:e01712-17. doi:10.1128/mBio.01712-17. 29362230 PMC5784249

[7] Khubaib M, Sheikh JA, Pandey S, Srikanth B, Bhuwan M, Khan N, Hasnain SE, Ehtesham NZ 2016 Mycobacterium tuberculosis co-operonic PE32/PPE65 proteins alter host immune responses by hampering Th1 response. Front Microbiol 7:719. doi:10.3389/fmicb.2016.00719. 27242739 PMC4868851

[8] Kohli S, Singh Y, Sharma K, Mittal A, Ehtesham NZ, Hasnain SE 2012 Comparative genomic and proteomic analyses of PE/PPE multigene family of Mycobacterium tuberculosis H(3)(7)Rv and H(3)(7)Ra reveal novel and interesting differences with implications in virulence. Nucleic Acids Res 40:7113–7122. doi:10.1093/nar/gks465. 22618876 PMC3424577

[9] Brennan MJ, Delogu G 2002 The PE multigene family: a “molecular mantra” for mycobacteria. Trends Microbiol 10:246–249. doi:10.1016/S0966-842X(02)02335-1. 11973159

[10] Tian C, Jian-Ping X 2010 Roles of PE_PGRS family in Mycobacterium tuberculosis pathogenesis and novel measures against tuberculosis. Microb Pathog 49:311–314. doi:10.1016/j.micpath.2010.07.004. 20638467

[11] Dheenadhayalan V, Delogu G, Brennan MJ 2006 Expression of the PE_PGRS 33 protein in Mycobacterium smegmatis triggers necrosis in macrophages and enhanced mycobacterial survival. Microbes Infect 8:262–272. doi:10.1016/j.micinf.2005.06.021. 16203168

[12] Chaitra MG, Shaila MS, Nayak R 2007 Evaluation of T-cell responses to peptides with MHC class I-binding motifs derived from PE_PGRS 33 protein of Mycobacterium tuberculosis. J Med Microbiol 56:466–474. doi:10.1099/jmm.0.46928-0. 17374885

[13] Chaitra MG, Shaila MS, Nayak R 2008 Detection of interferon gamma-secreting CD8(+) T lymphocytes in humans specific for three PE/PPE proteins of Mycobacterium tuberculosis. Microbes Infect 10:858–867. doi:10.1016/j.micinf.2008.04.017. 18653370

[14] Ramakrishnan L, Federspiel NA, Falkow S 2000 Granuloma-specific expression of Mycobacterium virulence proteins from the glycine-rich PE-PGRS family. Science 288:1436–1439. doi:10.1126/science.288.5470.1436. 10827956

[15] Campuzano J, Aguilar D, Arriaga K, León JC, Salas-Rangel LP, González-y-Merchand J, Hernández-Pando R, Espitia C 2007 The PGRS domain of Mycobacterium tuberculosis PE_PGRS Rv1759c antigen is an efficient subunit vaccine to prevent reactivation in a murine model of chronic tuberculosis. Vaccine 25:3722–3729. doi:10.1016/j.vaccine.2006.12.042. 17399860

[16] Brennan MJ, Delogu G, Chen YP, Bardarov S, Kriakov J, Alavi M, Jacobs WR 2001 Evidence that mycobacterial PE_PGRS proteins are cell surface constituents that influence interactions with other cells. Infect Immun 69:7326–7333. doi:10.1128/IAI.69.12.7326-7333.2001. 11705904 PMC98818

[17] Beatty WL, Ullrich HJ, Russell DG 2001 Mycobacterial surface moieties are released from infected macrophages by a constitutive exocytic event. Eur J Cell Biol 80:31–40. doi:10.1078/0171-9335-00131. 11211933

[18] Cadieux N, Parra M, Cohen H, Maric D, Morris SL, Brennan MJ 2011 Induction of cell death after localization to the host cell mitochondria by the Mycobacterium tuberculosis PE_PGRS33 protein. Microbiology 157:793–804. doi:10.1099/mic.0.041996-0. 21081760 PMC7336528

[19] Suragani M, Aadinarayana VD, Pinjari AB, Tanneeru K, Guruprasad L, Banerjee S, Pandey S, Chaudhuri TK, Ehtesham NZ 2013 Human resistin, a proinflammatory cytokine, shows chaperone-like activity. Proc Natl Acad Sci U S A 110:20467–20472. doi:10.1073/pnas.1306145110. 24282299 PMC3870731

[20] Lim YJ, Choi JA, Choi HH, Cho SN, Kim HJ, Jo EK, Park JK, Song CH 2011 Endoplasmic reticulum stress pathway-mediated apoptosis in macrophages contributes to the survival of Mycobacterium tuberculosis. PLoS One 6:e28531. doi:10.1371/journal.pone.0028531. 22194844 PMC3237454

[21] Seimon TA, Kim MJ, Blumenthal A, Koo J, Ehrt S, Wainwright H, Bekker LG, Kaplan G, Nathan C, Tabas I, Russell DG 2010 Induction of ER stress in macrophages of tuberculosis granulomas. PLoS One 5:e12772. doi:10.1371/journal.pone.0012772. 20856677 PMC2939897

[22] Choi HH, Shin DM, Kang G, Kim KH, Park JB, Hur GM, Lee HM, Lim YJ, Park JK, Jo EK, Song CH 2010 Endoplasmic reticulum stress response is involved in Mycobacterium tuberculosis protein ESAT-6-mediated apoptosis. FEBS Lett 584:2445–2454. doi:10.1016/j.febslet.2010.04.050. 20416295

[23] Choi JA, Lim YJ, Cho SN, Lee JH, Jeong JA, Kim EJ, Park JB, Kim SH, Park HS, Kim HJ, Song CH 2013 Mycobacterial HBHA induces endoplasmic reticulum stress-mediated apoptosis through the generation of reactive oxygen species and cytosolic Ca2+ in murine macrophage RAW 264.7 cells. Cell Death Dis 4:e957. doi:10.1038/cddis.2013.489. 24336077 PMC3877560

[24] Kruh NA, Troudt J, Izzo A, Prenni J, Dobos KM 2010 Portrait of a pathogen: the Mycobacterium tuberculosis proteome in vivo. PLoS One 5:e13938. doi:10.1371/journal.pone.0013938. 21085642 PMC2978697

[25] Lindestam Arlehamn CS, Gerasimova A, Mele F, Henderson R, Swann J, Greenbaum JA, Kim Y, Sidney J, James EA, Taplitz R, McKinney DM, Kwok WW, Grey H, Sallusto F, Peters B, Sette A 2013 Memory T cells in latent Mycobacterium tuberculosis infection are directed against three antigenic islands and largely contained in a CXCR3(+)CCR6(+) Th1 subset. PLoS Pathog 9:e1003130. doi:10.1371/journal.ppat.1003130. 23358848 PMC3554618

[26] Becq J, Gutierrez MC, Rosas-Magallanes V, Rauzier J, Gicquel B, Neyrolles O, Deschavanne P 2007 Contribution of horizontally acquired genomic islands to the evolution of the tubercle bacilli. Mol Biol Evol 24:1861–1871. doi:10.1093/molbev/msm111. 17545187

[27] Fontán P, Aris V, Ghanny S, Soteropoulos P, Smith I 2008 Global transcriptional profile of Mycobacterium tuberculosis during THP-1 human macrophage infection. Infect Immun 76:717–725. doi:10.1128/IAI.00974-07. 18070897 PMC2223452

[28] Hai TW, Liu F, Coukos WJ, Green MR 1989 Transcription factor ATF cDNA clones: an extensive family of leucine zipper proteins able to selectively form DNA-binding heterodimers. Genes Dev 3:2083–2090. doi:10.1101/gad.3.12b.2083. 2516827

[29] Ubeda M, Wang XZ, Zinszner H, Wu I, Habener JF, Ron D 1996 Stress-induced binding of the transcriptional factor CHOP to a novel DNA control element. Mol Cell Biol 16:1479–1489. doi:10.1128/MCB.16.4.1479. 8657121 PMC231132

[30] Liao Y, Fung TS, Huang M, Fang SG, Zhong Y, Liu DX 2013 Upregulation of CHOP/GADD153 during coronavirus infectious bronchitis virus infection modulates apoptosis by restricting activation of the extracellular signal-regulated kinase pathway. J Virol 87:8124–8134. doi:10.1128/JVI.00626-13. 23678184 PMC3700216

[31] Sano R, Reed JC 2013 ER stress-induced cell death mechanisms. Biochim Biophys Acta 1833:3460–3470. doi:10.1016/j.bbamcr.2013.06.028. 23850759 PMC3834229

[32] Bhandary B, Marahatta A, Kim HR, Chae HJ 2012 An involvement of oxidative stress in endoplasmic reticulum stress and its associated diseases. Int J Mol Sci 14:434–456. doi:10.3390/ijms14010434. 23263672 PMC3565273

[33] Hasnain SE, Taneja TK, Sah NK, Mohan M, Pathak N, Sahdev S, Athar M, Totey SM, Begum R 1999 In vitro cultured Spodoptera frugiperda insect cells: model for oxidative stress-induced apoptosis. J Biosci 24:13–19. doi:10.1007/BF02941101.

[34] Sah NK, Taneja TK, Pathak N, Begum R, Athar M, Hasnain SE 1999 The baculovirus antiapoptotic p35 gene also functions via an oxidant-dependent pathway. Proc Natl Acad Sci U S A 96:4838–4843. doi:10.1073/pnas.96.9.4838. 10220380 PMC21778

[35] Gotoh T, Mori M 2006 Nitric oxide and endoplasmic reticulum stress. Arterioscler Thromb Vasc Biol 26:1439–1446. doi:10.1161/01.ATV.0000223900.67024.15. 16645155

[36] Basu S, Pathak SK, Banerjee A, Pathak S, Bhattacharyya A, Yang Z, Talarico S, Kundu M, Basu J 2007 Execution of macrophage apoptosis by PE_PGRS33 of Mycobacterium tuberculosis is mediated by Toll-like receptor 2-dependent release of tumor necrosis factor-alpha. J Biol Chem 282:1039–1050. doi:10.1074/jbc.M604379200. 17095513

[37] Laskowski RA, Swindells MB 2011 LigPlot+: multiple ligand-protein interaction diagrams for drug discovery. J Chem Inform Model 51:2778–2786. doi:10.1021/ci200227u. 21919503

[38] Behar SM, Martin CJ, Booty MG, Nishimura T, Zhao X, Gan HX, Divangahi M, Remold HG 2011 Apoptosis is an innate defense function of macrophages against Mycobacterium tuberculosis. Mucosal Immunol 4:279–287. doi:10.1038/mi.2011.3. 21307848 PMC3155700

[39] Tundup S, Mohareer K, Hasnain SE 2014 Mycobacterium tuberculosis PE25/PPE41 protein complex induces necrosis in macrophages: role in virulence and disease reactivation? FEBS Open Bio 4:822–828. doi:10.1016/j.fob.2014.09.001. PMC4219985 25379378

[40] Fayyazi A, Eichmeyer B, Soruri A, Schweyer S, Herms J, Schwarz P, Radzun HJ 2000 Apoptosis of macrophages and T cells in tuberculosis associated caseous necrosis. J Pathol 191:417–425. doi:10.1002/1096-9896(2000)9999:9999<::AID-PATH664>3.0.CO;2-R. 10918217

[41] Pan H, Yan BS, Rojas M, Shebzukhov YV, Zhou H, Kobzik L, Higgins DE, Daly MJ, Bloom BR, Kramnik I 2005 Ipr1 gene mediates innate immunity to tuberculosis. Nature 434:767–772. doi:10.1038/nature03419. 15815631 PMC1388092

[42] Fulda S, Gorman AM, Hori O, Samali A 2010 Cellular stress responses: cell survival and cell death. Int J Cell Biol 2010:214074. doi:10.1155/2010/214074. 20182529 PMC2825543

[43] Zhang K, Kaufman RJ 2008 From endoplasmic-reticulum stress to the inflammatory response. Nature 454:455–462. doi:10.1038/nature07203. 18650916 PMC2727659

[44] Ehrt S, Schnappinger D 2009 Mycobacterial survival strategies in the phagosome: defence against host stresses. Cell Microbiol 11:1170–1178. doi:10.1111/j.1462-5822.2009.01335.x. 19438516 PMC3170014

[45] He S, Yaung J, Kim YH, Barron E, Ryan SJ, Hinton DR 2008 Endoplasmic reticulum stress induced by oxidative stress in retinal pigment epithelial cells. Graefes Arch Clin Exp Ophthalmol 246:677–683. doi:10.1007/s00417-008-0770-2. 18278507

[46] Estornes Y, Aguileta MA, Dubuisson C, De Keyser J, Goossens V, Kersse K, Samali A, Vandenabeele P, Bertrand MJ 2014 RIPK1 promotes death receptor-independent caspase-8-mediated apoptosis under unresolved ER stress conditions. Cell Death Dis 5:e1555. doi:10.1038/cddis.2014.523. 25476903 PMC4649839

[47] Jimbo A, Fujita E, Kouroku Y, Ohnishi J, Inohara N, Kuida K, Sakamaki K, Yonehara S, Momoi T 2003 ER stress induces caspase-8 activation, stimulating cytochrome c release and caspase-9 activation. Exp Cell Res 283:156–166. doi:10.1016/S0014-4827(02)00033-2. 12581736

[48] Tompa P 2002 Intrinsically unstructured proteins. Trends Biochem Sci 27:527–533. doi:10.1016/S0968-0004(02)02169-2. 12368089

[49] Via A, Uyar B, Brun C, Zanzoni A 2015 How pathogens use linear motifs to perturb host cell networks. Trends Biochem Sci 40:36–48. doi:10.1016/j.tibs.2014.11.001. 25475989

[50] Iantomasi R, Sali M, Cascioferro A, Palucci I, Zumbo A, Soldini S, Rocca S, Greco E, Maulucci G, De Spirito M, Fraziano M, Fadda G, Manganelli R, Delogu G 2012 PE_PGRS30 is required for the full virulence of Mycobacterium tuberculosis. Cell Microbiol 14:356–367. doi:10.1111/j.1462-5822.2011.01721.x. 22050772

[51] Chen T, Zhao Q, Li W, Xie J 2013 Mycobacterium tuberculosis PE_PGRS17 promotes the death of host cell and cytokines secretion via Erk kinase accompanying with enhanced survival of recombinant Mycobacterium smegmatis. J Interferon Cytokine Res 33:452–458. doi:10.1089/jir.2012.0083. 23663047 PMC3741429

[52] Huang Y, Zhou X, Bai Y, Yang L, Yin X, Wang Z, Zhao D 2012 Phagolysosome maturation of macrophages was reduced by PE_PGRS 62 protein expressing in Mycobacterium smegmatis and induced in IFN-γ priming. Vet Microbiol 160:117–125. doi:10.1016/j.vetmic.2012.05.011. 22658664

[53] Scull CM, Tabas I 2011 Mechanisms of ER stress-induced apoptosis in atherosclerosis. Arterioscler Thromb Vasc Biol 31:2792–2797. doi:10.1161/ATVBAHA.111.224881. 22096099 PMC3220876

[54] Salminen A, Ojala J, Kaarniranta K 2011 Apoptosis and aging: increased resistance to apoptosis enhances the aging process. Cell Mol Life Sci 68:1021–1031. doi:10.1007/s00018-010-0597-y. 21116678 PMC11114781

[55] Cambier CJ, O’Leary SM, O’Sullivan MP, Keane J, Ramakrishnan L 2017 Phenolic glycolipid facilitates mycobacterial escape from microbicidal tissue-resident macrophages. Immunity 47:552–565.e4. doi:10.1016/j.immuni.2017.08.003. 28844797 PMC5610147

[56] Harding CV, Boom WH 2010 Regulation of antigen presentation by Mycobacterium tuberculosis: a role for Toll-like receptors. Nat Rev Microbiol 8:296–307. doi:10.1038/nrmicro2321. 20234378 PMC3037727

[57] Stamm CE, Collins AC, Shiloh MU 2015 Sensing of Mycobacterium tuberculosis and consequences to both host and bacillus. Immunol Rev 264:204–219. doi:10.1111/imr.12263. 25703561 PMC4339209

[58] Mahajan S, Dkhar HK, Chandra V, Dave S, Nanduri R, Janmeja AK, Agrewala JN, Gupta P 2012 Mycobacterium tuberculosis modulates macrophage lipid-sensing nuclear receptors PPARγ and TR4 for survival. J Immunol 188:5593–5603. doi:10.4049/jimmunol.1103038. 22544925

[59] Kleinnijenhuis J, Oosting M, Joosten LAB, Netea MG, Van Crevel R 2011 Innate immune recognition of Mycobacterium tuberculosis. Clin Dev Immunol 2011:405310. doi:10.1155/2011/405310. 21603213 PMC3095423

[60] Mortaz E, Adcock IM, Tabarsi P, Masjedi MR, Mansouri D, Velayati AA, Casanova JL, Barnes PJ 2015 Interaction of pattern recognition receptors with Mycobacterium tuberculosis. J Clin Immunol 35:1–10. doi:10.1007/s10875-014-0103-7. PMC4306732 25312698

[61] Bansal K, Elluru SR, Narayana Y, Chaturvedi R, Patil SA, Kaveri SV, Bayry J, Balaji KN 2010 PE_PGRS antigens of Mycobacterium tuberculosis induce maturation and activation of human dendritic cells. J Immunol 184:3495–3504. doi:10.4049/jimmunol.0903299. 20176745

[62] Sánchez D, Rojas M, Hernández I, Radzioch D, García LF, Barrera LF 2010 Role of TLR2- and TLR4-mediated signaling in Mycobacterium tuberculosis-induced macrophage death. Cell Immunol 260:128–136. doi:10.1016/j.cellimm.2009.10.007. 19919859

[63] Tiwari B, Ramakrishnan UM, Raghunand TR 2015 The Mycobacterium tuberculosis protein pair PE9 (Rv1088)-PE10 (Rv1089) forms heterodimers and induces macrophage apoptosis through Toll-like receptor 4. Cell Microbiol 17:1653–1669. doi:10.1111/cmi.12462. 26031846

[64] Negi S, Pandey S, Srinivasan SM, Mohammed A, Guda C 2015 LocSigDB: a database of protein localization signals. Database 2015:bav003. doi:10.1093/database/bav003. 25725059 PMC4343182

[65] Grant CE, Bailey TL, Noble WS 2011 FIMO: scanning for occurrences of a given motif. Bioinformatics 27:1017–1018. doi:10.1093/bioinformatics/btr064. 21330290 PMC3065696

[66] UniProt Consortium 2017 UniProt: the universal protein knowledge base. Nucleic Acids Res 45:D158–D169. doi:10.1093/nar/gkw1099. 27899622 PMC5210571

[67] Yang J, Yan R, Roy A, Xu D, Poisson J, Zhang Y 2015 The I-TASSER suite: protein structure and function prediction. Nat Methods 12:7–8. doi:10.1038/nmeth.3213. 25549265 PMC4428668

[68] van Zundert GCP, Rodrigues JPGLM, Trellet M, Schmitz C, Kastritis PL, Karaca E, Melquiond ASJ, van Dijk M, de Vries SJ, Bonvin AMJJ 2016 The HADDOCK2.2 web server: user-friendly integrative modeling of biomolecular complexes. J Mol Biol 428:720–725. doi:10.1016/j.jmb.2015.09.014. 26410586

[69] Bhattacharya A, Tejero R, Montelione GT 2007 Evaluating protein structures determined by structural genomics consortia. Proteins 66:778–795. doi:10.1002/prot.21165. 17186527

[70] Abraham MJ, Van Der Spoel D, Lindahl E, Hess B 2014 The GROMACS development team. GROMACS user manual version 5.

[71] Pandey B, Grover S, Goyal S, Kumari A, Singh A, Jamal S, Kaur J, Grover A 2018 Alanine mutation of the catalytic sites of pantothenate synthetase causes distinct conformational changes in the ATP binding region. Sci Rep 8:903. doi:10.1038/s41598-017-19075-2. 29343701 PMC5772511

[72] Verma S, Singh A, Kumari A, Pandey B, Jamal S, Goyal S, Sinha S, Grover A 2018 Insight into the inhibitor discrimination by FLT3 F691L. Chem Biol Drug Des 91:1056–1064. doi:10.1111/cbdd.13169. 29336115

[73] Singh A, Grover S, Sinha S, Das M, Somvanshi P, Grover A 2017 Mechanistic principles behind molecular mechanism of rifampicin resistance in mutant RNA polymerase beta subunit of Mycobacterium tuberculosis. J Cell Biochem 118:4594–4606. doi:10.1002/jcb.26124. 28485504

[74] van Gunsteren WF, Daura X, Mark AE 2002 GROMOS force field. *In* von Ragué Schleyer P, Allinger NL, Clark T, Gasteiger J, Kollman PA, Schaefer HF, Schreiner PR (ed), Encyclopedia of computational chemistry, vol 2 John Wiley and Sons, Inc, Hoboken, NJ.

[75] Grover S, Arya R 2014 Role of UDP-N-acetylglucosamine-2-epimerase/N-acetylmannosamine kinase (GNE) in beta1-integrin-mediated cell adhesion. Mol Neurobiol 50:257–273. doi:10.1007/s12035-013-8604-6. 24474513

