# Supplementary figures and images for: The PGRS Domain of Mycobacterium tuberculosis PE_PGRS Protein Rv0297 Is Involved in Endoplasmic Reticulum Stress-Mediated Apoptosis through Toll-Like Receptor 4
Source: mBio. 2018 Jun 19;9(3):e01017-18. doi: 10.1128/mBio.01017-18 (PMC6016250; doi:10.1128/mBio.01017-18)

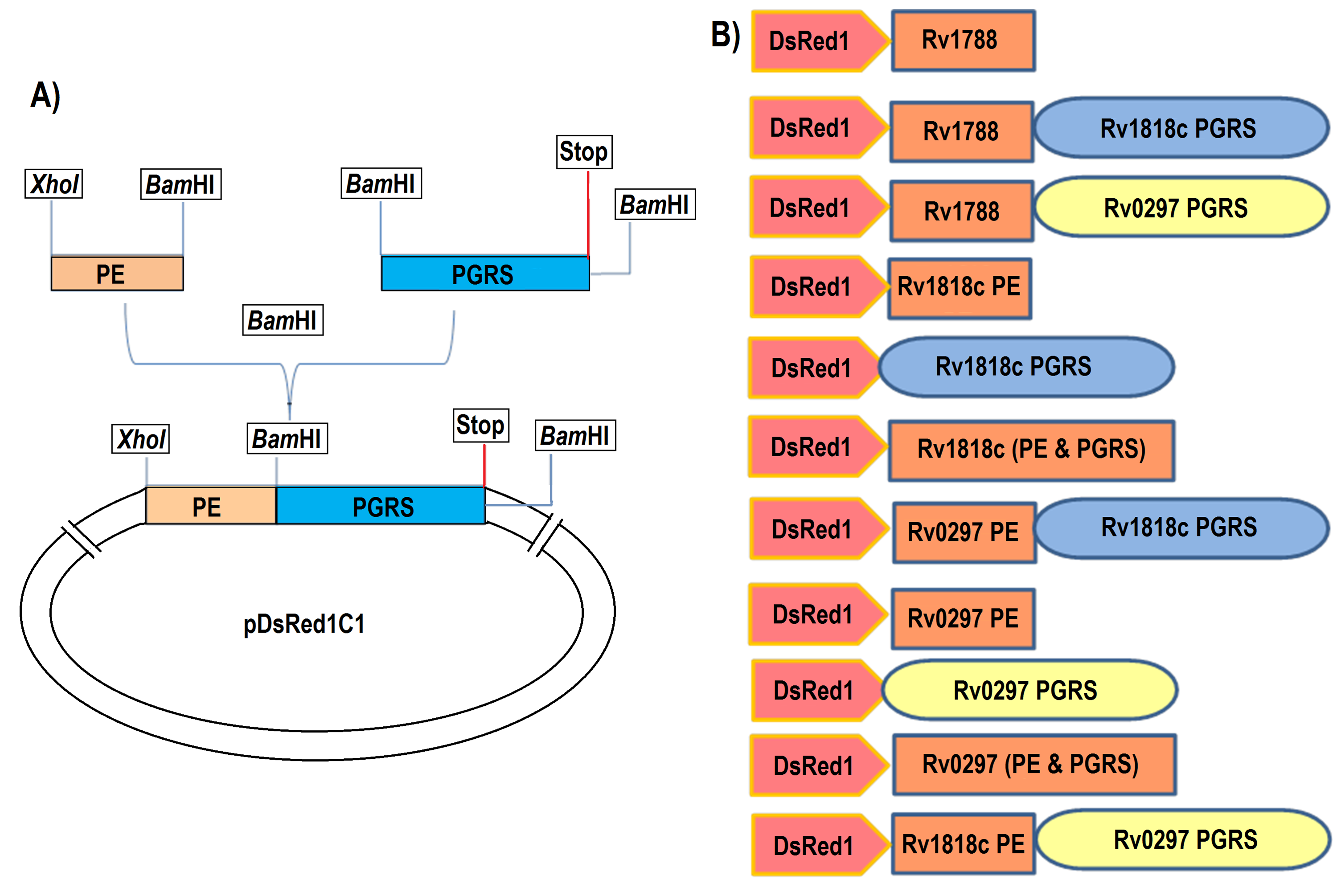

Supplement: FIG S1 [file mbo003183943sf1.tif]

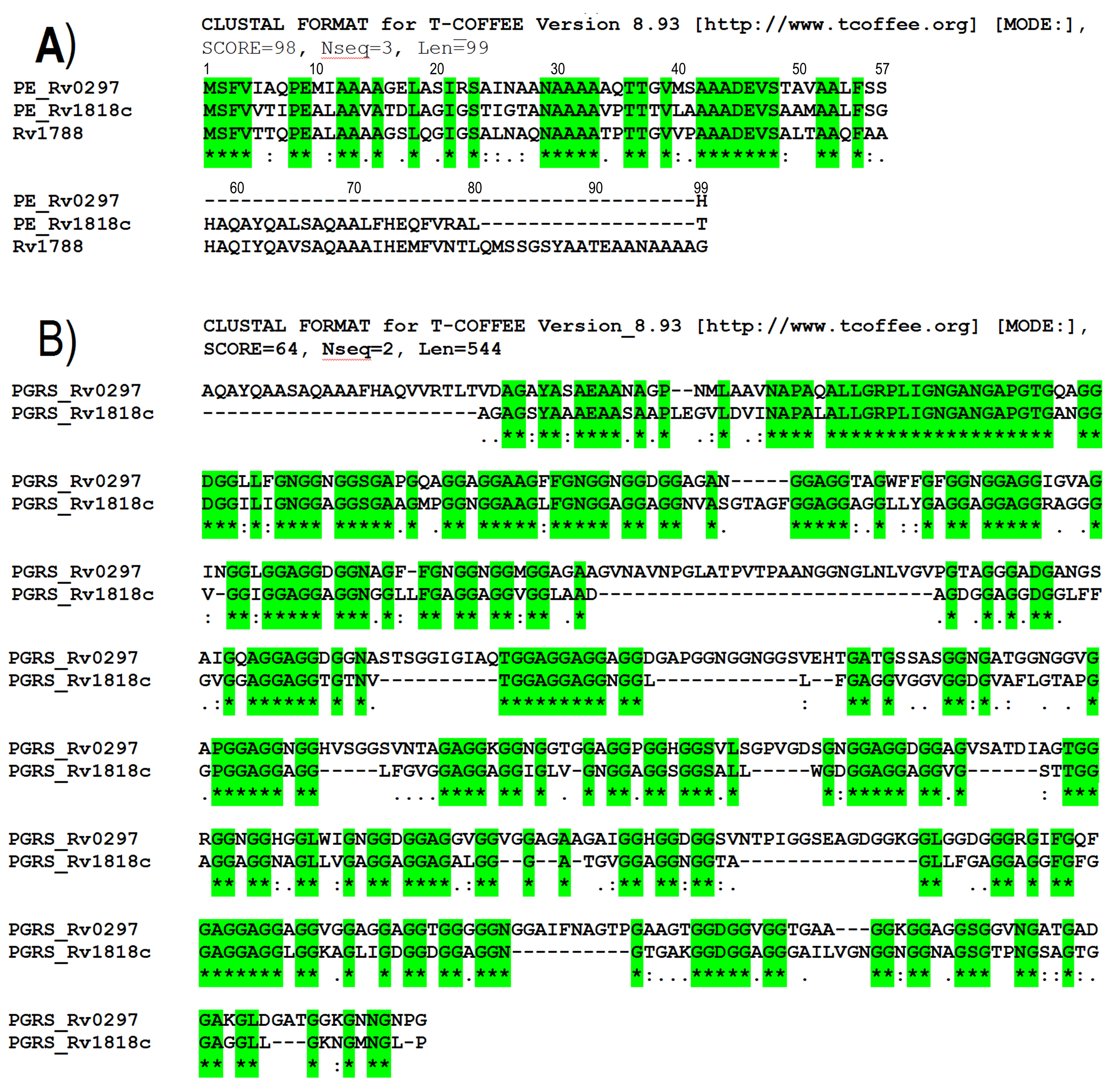

Supplement: FIG S2 [file mbo003183943sf2.tif]

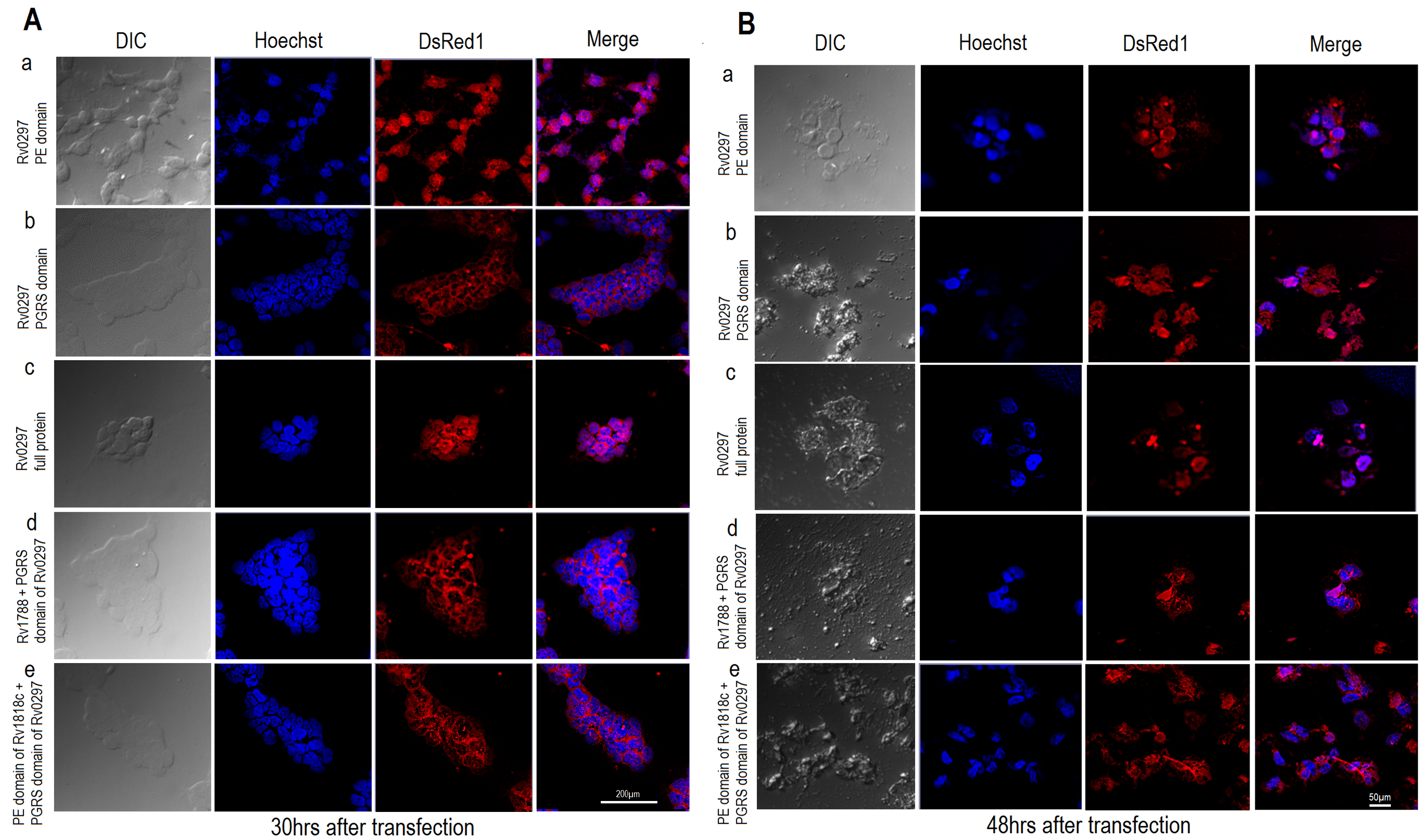

Supplement: FIG S3 [file mbo003183943sf3.jpg]

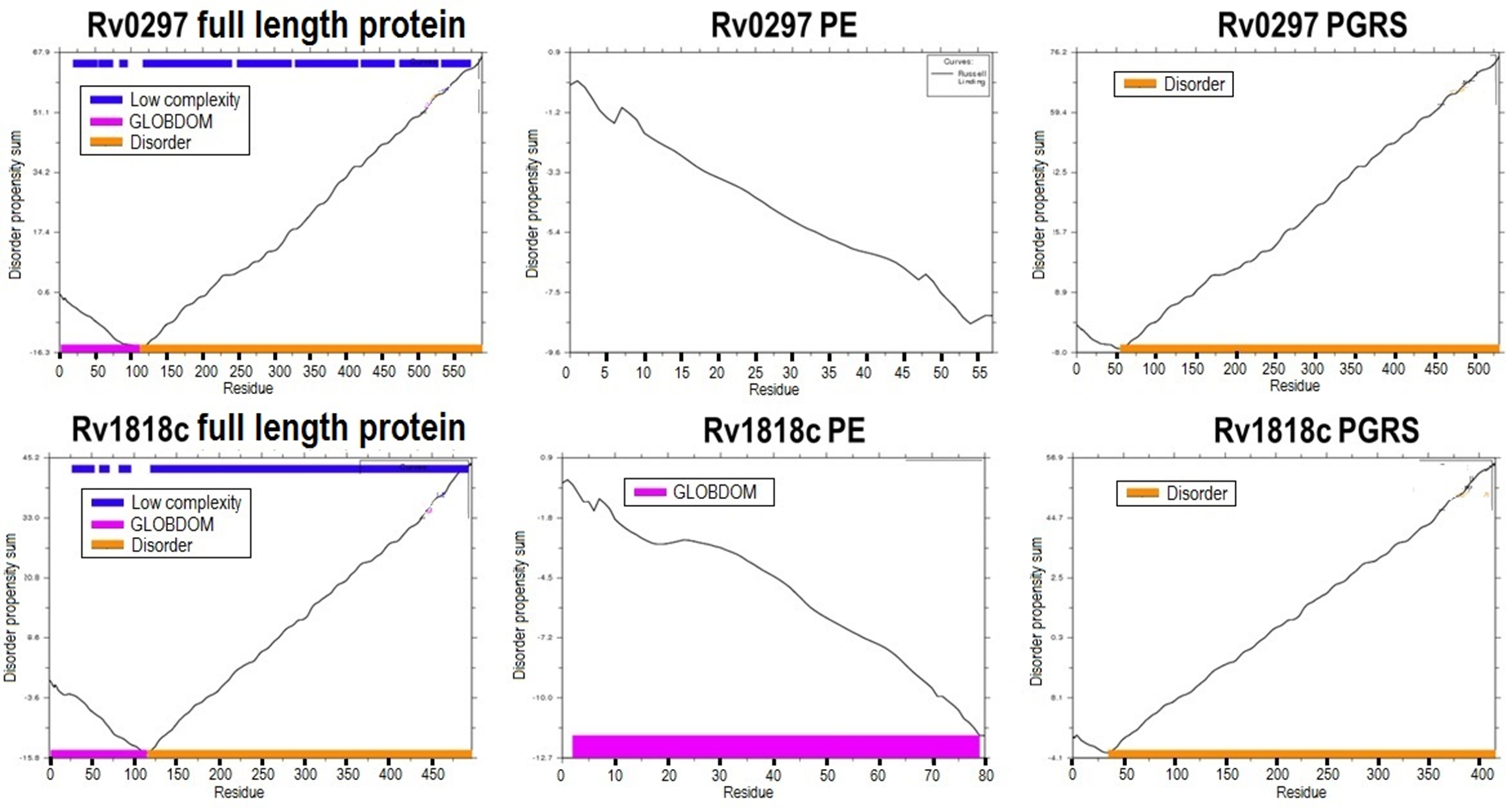

Supplement: FIG S4 [file mbo003183943sf4.tif]

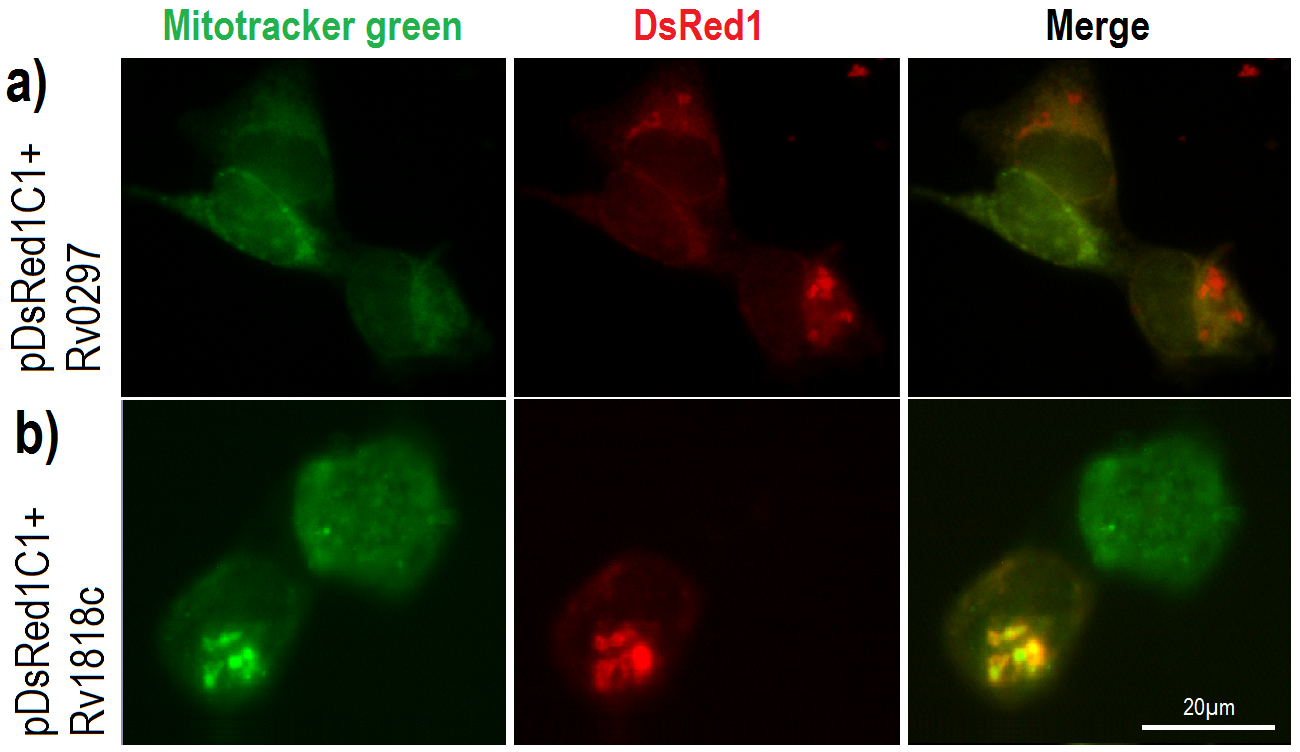

Supplement: FIG S5 [file mbo003183943sf5.tif]

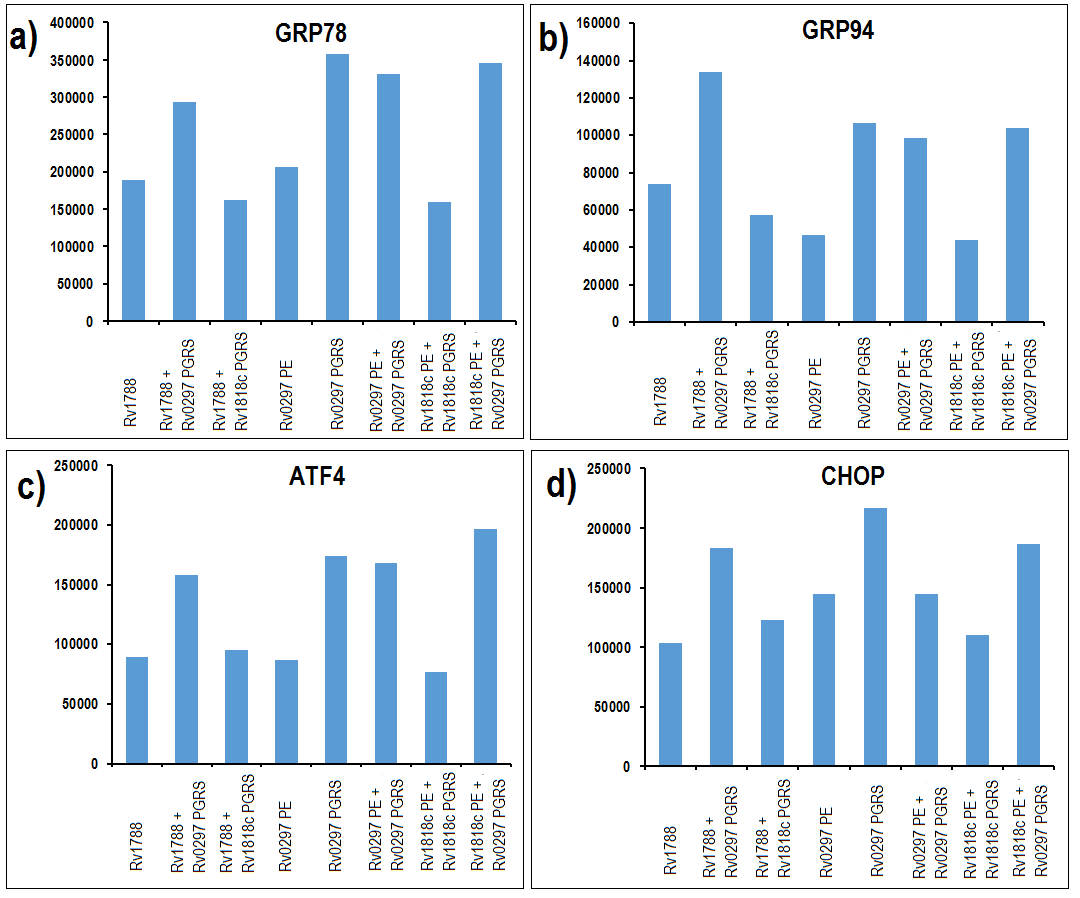

Supplement: FIG S6 [file mbo003183943sf6.tif]
