# Supplementary material for: The PGRS Domain of Mycobacterium tuberculosis PE_PGRS Protein Rv0297 Is Involved in Endoplasmic Reticulum Stress-Mediated Apoptosis through Toll-Like Receptor 4
Source: mBio. 2018 Jun 19;9(3):e01017-18. doi: 10.1128/mBio.01017-18 (PMC6016250; doi:10.1128/mBio.01017-18)
Supplement: TABLE S1 [file mbo003183943st1.docx]

**Table S1: Primer sequences**

| Primer name | Primer Sequence |
| --- | --- |
| Rv1818c Forward | AAACTCGAGCTATGTCATTTGTGGTC |
| Rv1818c Reverse | AAAGGATCCCTACGGTAACCCGTTC |
| Rv1818c PE Forward | AAACTCGAGCTATGTCATTTGTGGTC |
| Rv1818c PE Reverse | AAAAAGGATCCGGTGAGCGCCCG |
| Rv1818cPGRS forward | AAAAAGGATCCGGCTCGTATGCGG |
| Rv1818c PGRS Reverse | AAAGGATCCCTACGGTAACCCGTTC |
| Rv1788 Forward | AAAACTCGAGCTATGTCGTTTGTGACTACCCAAC |
| Rv1788 Reverse | AAAAAGGATCCCGAGCCGGCCGCG |
| Rv0297 Forward | AAAACTCGAGCTATGTCCTTTGTGATCGC |
| Rv0297 Reverse | AAAAAGGATCCTCAGCCGGGGTTGC |
| Rv0297 Reverse | AAAAAGGATCCATGCGAGGAAAACAGC |
| Rv0297 PGRS Forward | AAAAAGGATCCGCCCAGGCCTATCAG |
| Rv0297PGRS Forward (for pET28a) | AAAAAGGATCCGCCCAGGCCTATCAG |
| Rv0297 Forward (for pET28a) | AAAAAAAGCTTGCCGGGGTTGCCGTT |
