# Supplementary material for: The PGRS Domain of Mycobacterium tuberculosis PE_PGRS Protein Rv0297 Is Involved in Endoplasmic Reticulum Stress-Mediated Apoptosis through Toll-Like Receptor 4
Source: mBio. 2018 Jun 19;9(3):e01017-18. doi: 10.1128/mBio.01017-18 (PMC6016250; doi:10.1128/mBio.01017-18)
Supplement: TABLE S2 [file mbo003183943st2.docx]

**Table S2: PCR conditions for the amplicons.**

| Rv1818c PE  1: 95°C            300 sec  2: 95°C            30 sec  3: 49°C            30 sec  4: 72°C            30 sec  5: Repeat 2 to 4 –     6 times      (-1.0°C/cycle)  6: 95°C            30 sec  7: 59°C            30 sec  8: 72°C            30 sec  9: Repeat 6 to 8 –     30 times  10: 72°C          10 min | Rv1818c PGRS  1: 95°C            300 sec  2: 95°C            30 sec  3: 54°C            30 sec  4: 72°C            90 sec  5: Repeat 2 to 4 –     6 times      (-1.0°C/cycle)  6: 95°C            30 sec  7: 61°C            30 sec  8: 72°C            90 sec  9: Repeat 6 to 8 –     30 times  10: 72°C          10 min | Rv1818c  1: 95°C           300 sec  2: 95°C             30 sec  3: 47°C             30 sec  4: 72°C           105 sec  5: Repeat 2 to 4 –     4 times      (-0.5°C/cycle)  6: 95°C             30 sec  7: 57°C             30 sec  8: 72°C           105 sec  9: Repeat 6 to 8 –     30 times  10: 72°C          10 min |
| --- | --- | --- |
| Rv1788    1: 95°C            300 sec  2: 95°C            30 sec  3: 56°C            30 sec  4: 72°C            30 sec  5: 95°C            30 sec  6: 65°C            30 sec  7: 72°C            30 sec  8: Repeat 5 to7 – 33 times  9: 72°C            10 min | Rv0297 PE  1: 95°C            300 sec  2: 95°C            30 sec  3: 50°C            30 sec  4: 72°C            30 sec  5: 95°C            30 sec  6: 62°C            30 sec  7: 72°C            30 sec  8: Repeat 5 to7 – 33 times  9: 72°C            10 min | Rv0297, Rv0297 PGRS  1: 95°C            300 sec  2: 95°C            30 sec  3: 51°C            30 sec  4: 72°C            110 sec  5: 95°C            30 sec  6: 64°C            30 sec  7: 72°C            110 sec  8: Repeat 5 to7 – 33 times  9: 72°C            10 min |
