# Supplementary material for: The PGRS Domain of Mycobacterium tuberculosis PE_PGRS Protein Rv0297 Is Involved in Endoplasmic Reticulum Stress-Mediated Apoptosis through Toll-Like Receptor 4
Source: mBio. 2018 Jun 19;9(3):e01017-18. doi: 10.1128/mBio.01017-18 (PMC6016250; doi:10.1128/mBio.01017-18)
Supplement: TABLE S4 [file mbo003183943st4.docx]

Table S4: Comparison of Pre-MD and Post-MD interactions of TLR4-Rv0297 complex.

| **Interacting residues** | **TLR4 Pre-MD** | **TLR4 Post-MD** | **Rv0297 Pre-MD** | **Rv0297 Post-MD** |
| --- | --- | --- | --- | --- |
| **Hydrogen Bonding Interactions** | Arg289, Ser360, Lys402, Tyr403, **Glu425**, **Asp428**, Gln430, **His431**, Tyr451, **Lys477**, **Asp502**, **Ser504**, **Asn526**, Ser528, **Asp550**, **Asn575** | Arg234, Glu262, Arg289, Ala291, Ser360, Arg382, **Glu425**, **Asp428**, **His431**, **Lys477**, **Asp502**, **Ser504**, Gln505, **Asn526**, Gln547, **Asp550**, **Asn575**, Glu603 | Met1, Phe3, Gu17, **Ser41**, Ala43, **Ala93**, Pro124, **Thr126**, **Glu128**, Ala129, **Gly131**, Gly183, Asn224, Gly583, **Lys584**, Asn587 | Val4, Glu9, Gly16, Leu18, Ala19, Ser20, Ala42, **Ser41**, **Ala93,** Gly119, Gly122, **Thr126, Gln128**, GLY130, **Gly131**, Asn142, Asn163, Asn224, Thr581, **Lys584** |
| **Hydrophobic Interactions** | Glu336, Val338, Thr359, Phe377, **Ser381**, Arg382, Ser407, **Phe408**, **His426**, **Ile450**, **Glu474**, **Thr499**, **Phe500**, Glu505, His529, Val548, **Phe573** | Val259, Gly261, Phe263, Tyr292, Asn339, Lys362, **Ser381**, **Phe408**, **His426**, Gln430, **Ile450**, Tyr451, Asp453, **Glu474**, Val475, **Thr499**, **Phe500**, Ser528, Leu553, **Phe573**, Thr577 | Ser2, Val4, **Ile5**, Met10, Asp45 **Asn94**, **Trp184**, **Phe185**, Phe186, **Leu206**, **Gly207**, Gly411, **Gly582** | Phe3, **Ile5**, Glu17, **Asn94**, Ala120, Asn121, Ala123, Pro124, **Trp184**, **Phe185**, **Leu206**, **Gly207,** Met227, Asp410, **Gly582**, Gly583, Asn587 |
